# Characterization of Broad Spectrum Bacteriophage vB ESM-pEJ01 and Its Antimicrobial Efficacy Against Shiga Toxin-Producing *Escherichia coli* in Green Juice

**DOI:** 10.3390/microorganisms13010103

**Published:** 2025-01-07

**Authors:** Eun Jeong Park, Seungki Lee, Jong Beom Na, Ye Bin Kim, Kee Man Lee, Seon Young Park, Ji Hyung Kim

**Affiliations:** 1 Department of Food Science and Biotechnology, College of Bionano Technology, Gachon University, Seongnam 13120, Republic of Korea; pog0227@gachon.ac.kr (E.J.P.); qwer789@gachon.ac.kr (J.B.N.); zxc5620@gachon.ac.kr (Y.B.K.); 99angello@gachon.ac.kr (K.M.L.); 2 Biological and Genetic Resources Assessment Division, National Institute of Biological Resources, Incheon 22689, Republic of Korea; metany@korea.kr; 3 Laboratory of Aquatic Biomedicine, College of Veterinary Medicine and Research Institute for Veterinary Science, Seoul National University, Seoul 08826, Republic of Korea

**Keywords:** Shiga toxin-producing *Escherichia coli* (STEC), polyvalent phage, biocontrol, food application, green juice

## Abstract

Shiga toxin-producing *Escherichia coli* (STEC) infections have increased in humans, animals, and the food industry, with ready-to-eat (RTE) food products being particularly susceptible to contamination. The prevalence of multidrug-resistant strains has rendered the current control strategies insufficient to effectively control STEC infections. Herein, we characterized the newly isolated STEC phage vB_ESM-pEJ01, a polyvalent phage capable of infecting *Escherichia* and *Salmonella* species, and assessed its efficacy in reducing STEC in vitro and food matrices. The phage, belonging to the *Tevenvirinae*, exhibits effective bacteriolytic activity, a short latent period, large burst size, and stability under a broad pH range and moderate temperatures. Moreover, the phage demonstrated strong anti-biofilm efficacy even at low concentrations. Genomic analysis revealed that the phage was similar to the well-characterized RB49 phage (T4-like phage) but possesses distinct host-specificity-related genes that potentially contribute to its extensive host range. The efficacy of phage vB_ESM-pEJ01 was evaluated in artificially STEC-inoculated green juice samples, where it significantly reduced STEC and the abundance of Shiga toxin-producing genes at 4 and 25 °C. Therefore, these results suggest that the polyvalent phage vB_ESM-pEJ01 is a promising biocontrol agent for foodborne pathogens in RTE foods such as fresh juices.

## 1. Introduction

Pathogenic *Escherichia* (*E*.) *coli* infections have increased in humans, animals, and the food industry, with Shiga toxin-producing *E*. *coli* (STEC) being the most well-known foodborne pathogen [1]. STEC infections can cause severe symptoms, such as bloody diarrhea, hemorrhagic colitis, and hemolytic uremic syndrome [1]. The primary route of STEC transmission to humans is the ingestion of contaminated foods, including undercooked ground beef and unpasteurized spinach [2,3]. Recently, the increasing demand for ready-to-eat (RTE) food products, such as fresh produce and juices, has coincided with a notable rise in STEC outbreaks linked to these products worldwide [4,5]. RTE products are particularly susceptible to STEC contamination during packaging, transportation, and storage [6]. Despite adopting control measures, current strategies have proven ineffective in eliminating the pathogen, highlighting the growing importance of RTE products as a significant source of STEC infections [5]. Although antimicrobials are commonly used to prevent and treat STEC infections, excessive and improper use has led to the emergence of multidrug-resistant STEC strains [7,8]. Therefore, discovering alternative antimicrobial agents, such as bacteriophages (phages), has become increasingly crucial for controlling STEC in food products.

Phages are viruses that infect and lyse bacterial cells, making them promising alternatives to antimicrobials to ensure food safety and efficacy [9]. Several studies have demonstrated the successful use of phages for the biocontrol of STEC in various food matrices, such as burger patties, lettuce, and tomatoes [10,11]. The US Food and Drug Administration (FDA) has approved several commercial phage-based products for use in food-processing facilities to significantly reduce foodborne pathogens. In 2012, EcoShield^TM^ (Intralytix, Inc., Baltimore, MD, USA) was approved to control *E*. *coli* [10], followed by the approval of SalmoFresh^TM^ (Intralytix Inc., USA) in 2013 for reducing pathogenic *Salmonella* [12]. Moreover, the FDA granted the “Generally Recognized as Safe” status to phage-based products targeting foodborne pathogens, including STEC, allowing their use as food additives with no pre-market review when used under “Good Manufacturing Practices” [13].

One of the critical advantages of phages as biocontrol agents is their high specificity for targeting host bacteria, allowing for the selective lysis of pathogenic bacteria [9]. However, this can be limited when faced with threats from multiple pathogenic bacteria. To overcome this limitation of phage specificity, several studies have used phage cocktails to control different bacterial species and serotypes, including those infecting *E. coli* and *Salmonella* serovar Typhimurium [14,15]. While phage cocktails have been used to address the limited host range of individual phages, their preparation presents significant challenges, such as compatible-specific phage selection and high manufacturing costs. An alternative approach to overcoming these challenges is the identification of broad-host-range phages, which can simultaneously infect multiple bacterial species or genera, potentially eliminating the need for complex phage cocktail formulations [16]. Among these phages, those capable of infecting more than two bacterial genera are currently categorized as polyvalent phages [17]. The use of polyvalent phages could potentially simplify the formulation process and replace intricate phage cocktails, offering a more scalable and cost-effective approach. Several studies have reported the isolation of polyvalent phages that infect the *Enterobacteriaceae* family [18,19,20]. These have significant potential for industrial applications as a balanced strategy that ensures effective biocontrol while preserving the ecological functioning of microbial communities, offering a clear advantage over phage cocktails, which may disrupt microbial ecosystems [17,21]. Despite the potential advantages of polyvalent phages, reports on their isolation and characterization of polyvalent phages remain limited. Considering the potential application of polyvalent phages in controlling pathogenic bacteria, such as STEC, in food products, it is imperative to evaluate their stability and efficacy under various environmental conditions to ensure their suitability as biocontrol agents.

Therefore, the objectives of this study were to (1) characterize the newly isolated STEC phage vB_ESM-pEJ01, focusing on its biological and genomic properties as a polyvalent phage, (2) evaluate the efficacy of its potential application in STEC-contaminated food products. These findings would provide valuable insights into the potential of polyvalent phages for controlling STEC contamination and enhancing food safety, informing future phage-based biocontrol strategies.

## 2. Materials and Methods

### 2.1. Phage Isolation and Host Range Determination

The phage was isolated from wastewater collected from a sewage treatment plant in Daejeon, using STEC ATCC 43895 as a host bacterial strain, as previously described [22]. A 1 mL of overnight bacterial culture in tryptic soy broth (TSB; Difco, Detroit, MI, USA) was mixed with a sewage sample and incubated at 37 °C with shaking. The mixture was centrifuged at 10,000× *g* for 20 min to remove the bacterial pellet, and the supernatant was filtered through a 0.22 µm membrane filter (Millipore, Burlington, MA, USA) to secure phages from the mixture. A presumptive phage plaque was isolated on 0.7% tryptic soy agar (TSA; Difco, USA) using the double-layer agar method, repeated at least three times until a single plaque was observed on bacterial lawns. The isolated phage lysates were stored in TSB with 10% glycerol at −80 °C. The host spectrum of the phage was conducted using 26 strains of *Escherichia* spp. (including 15 STEC, 7 non-STEC, 2 enterohemorrhagic *E*. *coli*, *E*. *hermanni*, and *E*. *fergusonii*) and 45 strains of *Salmonella enterica* serovars (including 28 Enteritidis, 10 Typhimurium, and 1 each of Agona, Bareilly, Infantis, Senftenberg, Motevideo, Java, and Heidelberg). After spotting 10 µL of phage lysate (~10^7^ PFU/mL) onto the layer of each bacterial strain on TSA plates, clear plaques were evaluated based on PFU counting.

### 2.2. Transmission Electron Microscopy (TEM) Analysis

To examine the morphology of the isolated STEC phage, the phage lysate (~10^8^ PFU/mL) was placed on glow-discharged carbon-coated copper grids and subjected to negative staining with 2% (*w*/*v*) uranyl acetate (Electron Microscopy Sciences Inc., Hatfield, PA, USA). The grids were observed under a transmission electron microscope (JEM-1400 Plus, JEOL Ltd., Tokyo, Japan) at 120 kV at the Korea Basic Science Institute (Cheongju, Republic of Korea). The resulting electron microscopy images were analyzed using ImageJ software (https://imagej.net/ij, accessed on 15 June 2024) [23].

### 2.3. Bacteriolytic Activity

The bacteriolytic activity of the isolated STEC phage was assessed using the host bacterial STEC ATCC 43895. A 1% inoculum of an overnight bacterial culture (~10^7^ CFU/mL) was introduced into 40 mL of fresh TSB to facilitate the exponential growth phase of the bacterial strain and added to the phage suspension at a multiplicity of infection (MOI) of 0.001, 0.01, 0.1, 1, 10, and 100. The bacterial strain was inoculated into TSB as a positive control, and diluted water was used as a negative control. Each mixture was inoculated in 200 µL into a 96-well plate, repeated more than at least three times. Samples of the mixture were taken at hourly intervals up to 6 h for incubation at 37 °C. The optical density of the bacteria was measured at OD_600_nm using a UV/Vis spectrophotometer (SpectraMax, Molecular Devices, San Jose, CA, USA).

### 2.4. Adsorption and One-Step Growth

Adsorption and one-step growth assays of the isolated STEC phage were performed as previously described [24]. For the adsorption assay, the phage suspension was mixed with the host bacterial strain (~10^7^ CFU/mL) at an MOI of 0.01 and incubated at 37 °C with shaking. At 5 min intervals of up to 15 min, 100 µL samples of the mixture were collected. These samples were then filtered using a 0.22 µm membrane filter to obtain the un-adsorbed free phage, and serial dilutions of the filtrate were performed for PFU enumeration, considering the initial count as the baseline for 100% un-adsorbed phages. For the one-step growth assay, the phage suspension was mixed with the host bacterial strain (~10^7^ CFU/mL) at an MOI of 0.001 and incubated at 37 °C for 10 min, a time point by which the adsorption rate of the phage exceeded 90%. The mixture was centrifuged at 10,000× *g* for 5 min, the supernatant was discarded, and the bacterial pellet was collected at intervals ranging from 0 to 90 min. Subsequently, PFU counts were performed at these time points to determine the latent time and burst size of the phage.

### 2.5. Thermal and pH Stability

Thermal and pH stability assays of the isolated STEC phage were performed as described previously [22]. To determine the sensitivity of the phage to various environmental conditions, the phage lysate (~10^6^ PFU/mL) was tested by exposing it to a range of temperatures (4, 25, 37, 56, and 80 °C) and pH levels (3, 4, 5, 6, 7, 8, 9, 10, and 11). For these stability assays, 1 mL of the phage suspension was adjusted to the aforementioned temperatures for thermal testing and to different pH values at 37 °C for pH testing. After 3 h of incubation under both sets of conditions, the phage titer (PFU/mL) was determined through serial dilution using the double-layer agar method.

### 2.6. Anti-Biofilm Ability

The ability of the isolated STEC phage to prevent bacterial biofilm formation was assessed as described previously [25]. A 1% inoculum of an overnight bacterial culture (~10^7^ CFU/mL) was diluted into 40 mL of fresh TSB, and 100 µL of this bacterial dilution was mixed with 100 µL phage suspension at different MOIs ranging from 0.000001 to 1. The resultant 200 µL mixtures were inoculated into each well of a 96-well plate and then incubated at 37 °C for 24 and 48 h. Following incubation, the supernatants were discarded, and each well was washed twice with diluted water to retain only the biofilm. The biofilms were then stained with 0.1% crystal violet (CV) at room temperature for 20 min, followed by washing and dissolution in 95% ethanol at room temperature for another 20 min. The total biomass of the biofilm was quantified by measuring at OD_595_ nm using a UV/Vis spectrophotometer.

### 2.7. Whole-Genome Sequencing and Bioinformatics Analysis

The genomic DNA of the isolated STEC phage was extracted using a Phage DNA Isolation Kit (Norgen Bioteck Corp., Thorold, ON, Canada). Following extraction, a DNA library was prepared using the TruSeq Nano DNA Library Prep Kit (Illumina, San Diego, CA, USA), sequenced on the Illumina HiSeq X-10 platform (Illumina, USA), and de novo assembly was performed using SPAdes v3.4.1. [26]. The open reading frames (ORFs) in the assembled phage genome were annotated using the Rapid Annotation from Subsystem Technology (RAST) server (https://rast.nmpdr.org, accessed on 21 February 2024) [27], and putative functions of ORFs were predicted with BLASTP (https://blast.ncbi.nlm.nih.gov/Blast.cgi, accessed on 1 March 2024), InterPro 97.0. (https://www.ebi.ac.uk/interpro/, accessed on 1 March 2024) [28], and Pfam database (http://pfam.xfam.org/, accessed on 1 March 2024) [29]. The transmembrane domains and signal peptides were predicted using DeepTMHMM v.1.0.24. (https://dtu.biolib.com/DeepTMHMM, accessed on 1 March 2024) [30] and SignalP v.6.0. (https://services.healthtech.dtu.dk/services/SignalP-6.0/, accessed on 1 March 2024) [31]. Additionally, the phage genome was analyzed for the presence of tRNAs, virulence factors, and antibiotic resistance genes using tRNAscan-SE v.2.0. (http://gtrnadb.ucsc.edu/faq.html, accessed on 11 March 2024) [32], Virulence Factors of Pathogenic Bacteria Database (VFDB; http://www.mgc.ac.cn/VFs/, accessed on 11 March 2024) [33], and Antibiotic Resistance Gene-ANNOTation (https://www.mediterranee-infection.com, accessed on 11 March 2024) [34], respectively. The packaging system and genome termini were determined using PhageTerm (https://galaxy.pasteur.fr, accessed on 16 March 2024) [35]. A circular genome map of the isolated STEC phage was illustrated using the CGView server (http://cgview.ca/, accessed on 7 April 2024) [36]. The genomic phylogenetic analysis of the isolated STEC phage, based on the classification standard proposed by ICTV (https://ictv.global/taxonomy/, accessed on 10 April 2024), was constructed using phages belonging to 18 different genera within the *Straboviridae* family available in GenBank and was executed using VICTOR (https://victor.dsmz.de, accessed on 1 August 2024) [37]. Maximum-likelihood phylogenetic trees were generated using amino acid sequences of the terminase large subunit and major capsid protein using MEGA 11 software (http://www.megasoftware.net/, accessed on 10 April 2024) [38] with 1000 bootstrap replicates. Additionally, genomic comparison of the isolated STEC phage was performed with six *Straboviridae* family phages, including *Enterobacteria* phage RB49 (NC_005066), *Escherichia* phage ECD7 (NC_041936), *Escherichia* virus KFS-EC (NC_055757), *Enterobacteria* phage JSE (NC_012740), *Enterobacteria* phage Phi1 (NC_009821), and *Enterobacteria* phage GEC-3S (NC_025425). The nucleotide intergenomic similarity between the isolated STEC phage and six representative phages was assessed using VIRIDIC (http://viridic.icbm.de, accessed on 12 April 2024) [39], and their comparative genomics was visualized using ViPTree (https://www.genome.jp/viptree, accessed on 12 April 2024) [40].

### 2.8. Evaluation of Phage Biocontrol Efficacy Against STEC-Contaminated Green Juice

The biocontrol efficacy of the isolated phage in reducing STEC contamination in food products was assessed using green juice purchased from a local market in Korea. Before the experiment, green juice samples were sterilized by exposure to UV light under a hood for 1 h at room temperature. The absence of microorganisms in the sterilized juice was confirmed by plating on TSA before artificially inoculating STEC. A 100 µL of overnight STEC ATCC 43895 culture (~10^7^ CFU/mL) was diluted in 10 mL fresh TSB and inoculated into 10 mL green juice. The mixture was subsequently treated with 10 mL of the phage suspension to reach an MOI of 0.1. Samples were only inoculated with the bacterial strain as a positive control and only with diluted water as a negative control. The samples were incubated at both 4 and 25 °C, respectively, and collected at intervals of 0, 3, 6, 9, 12, and 24 h post-inoculation. The number of STEC in the samples was determined through serial dilutions by plating on CHROMagar STEC (CHROMagar Microbiology, Paris, France) to quantify the bacterial count. To evaluate the abundance of Shiga toxin-producing genes in the collected samples, gDNA was extracted using a DNeasy Blood & Tissue Kit (QIAGEN, Hilden, Germany). The gDNA extracted from each sample served as a template for quantifying the presence of *stx* genes using real-time quantitative PCR (qPCR) with two primer sets [41], targeting *stx*1 and *stx*2 genes (Table 1). Each qPCR reaction mixture comprised 10 μL of 2X QGreenBlue qPCR Master Mix (CellSafe, Yongin, Republic of Korea), 1 μL of sample DNA, 1 μL of both forward and reverse primers, and 7 μL of diethylpyrocarbonate (DEPC), making a total volume of 20 μL. The qPCR was conducted in more triplicates for each sample using a QuantStudio^TM^ 1 Real-Time PCR system (ThermoFisher Scientific, Waltham, MA, USA) under the following conditions: initial denaturation at 95 °C for 10 min, followed by 40 cycles of denaturation at 95 °C for 30 s, annealing at 55 °C for 1 min, and extension at 72 °C for 30 s. For absolute quantification, the standard curve was generated for each *stx* gene using DNA extracted STEC ATCC 43895, containing DNA copies ranging from 10^2^ to 10^6^ per µL. DNA concentration was measured using a Nanodrop ND-1000 spectrophotometer (Thermo Fisher Scientific, Waltham, MA, USA), and copy numbers were calculated using the Copy Number Calculator tool (https://www.technologynetworks.com/tn/tools/copynumbercalculator, accessed on 15 February 2024). The amplification efficiency of qPCR was estimated based on the slope of the standard curve, considering its values between 95 and 105%.

### 2.9. Statistical Analysis

Statistical analysis was performed through a one-way analysis of variance (ANOVA), complemented by a Bonferroni post-hoc test for detailed comparisons, and the analysis was conducted using the SPSS software v28.0.0.0 (SPSS Inc., Richmond, Chicago, IL, USA). A *p* < 0.05 was set to determine statistical significance.

## 3. Results

### 3.1. Morphology of STEC Phage vB_ESM-pEJ01

**Figure 1:**
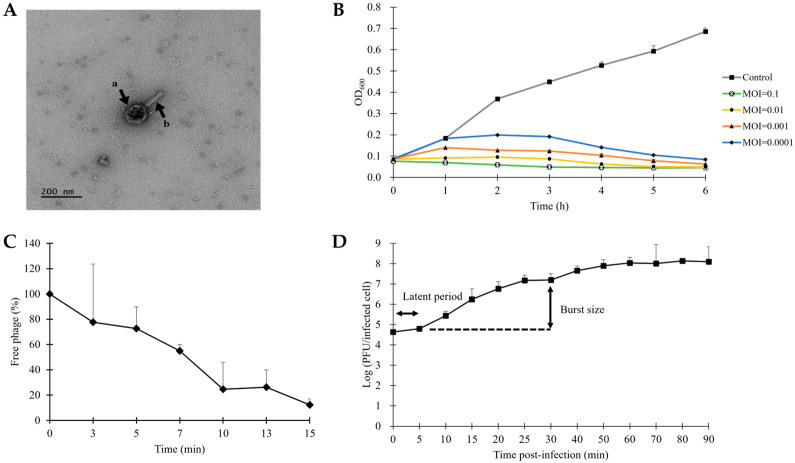
Biological characteristics of STEC phage vB_ESM-pEJ01. (**A**) Transmission electron micrograph of the phage is represented by the black arrows a and b with the icosahedral head and the contracted tail, respectively. The scale bar is 200 nm. (**B**) The bacteriolytic activity of the phage was assessed at four different MOIs (0.0001, 0.001, 0.01, and 0.1) on host strain ATCC 43895. The kinetics of adsorption rate (**C**) and one-step growth (**D**) of the phage. The adsorption rate was evaluated by calculating the percentage of free phages at an MOI of 0.01. The one-step growth curve depicted the latent time and burst size of the phage. Error bars represent the standard deviation of three replicates.

The newly isolated STEC phage, designated vB_ESM-pRJ01 following the guidelines for bacteriophage nomenclature [42], was isolated from a sewage sample using STEC ATCC 43895 as the host strain. TEM analysis revealed that the phage possesses a *Myoviridae* morphotype, showing an icosahedral head with a 101.1 ± 0.1 nm diameter and a contractile tail with 115. 3 ± 0.3 nm in length (Figure 1A).

### 3.2. Host Range of STEC Phage vB_ESM-pEJ01

The host range of the STEC phage vB_ESM-pEJ01 was determined by conducting spot assays using 26 Escherichia species and 45 serovars of *S*. *enterica* subsp. *enterica*, with clear plaque formation indicating host infectivity (Table 2). The phage efficiently lysed 14 of the 15 STEC strains and all non-STEC strains of Enterohemorrhagic *E. coli* NCCP 13721, *E*. *hermanni* ATCC 33650^T^, and *E*. *fergusonii* ATCC 35469^T^, demonstrating its broad host range within the *Escherichia* genus. Furthermore, the phage was tested using multiple serovars of *S*. *enterica* subsp. *enterica*, including Enteritidis, Typhimurium, Agona, Bareilly, Infantis, Senftenberg, Motevideo, Java, and Heidelberg, and efficiently lysed 10 of 28 *S*. Enteritidis strains, seven of 10 *S*. Typhimurium strains, and one strain each of *S*. Agona, *S*. Java, and *S*. Heidelberg.

### 3.3. Bacteriolytic Activity of STEC Phage vB_ESM-pEJ01

To evaluate the bacteriolytic activity of phage vB_ESM-pEJ01, the growth kinetic of its host strain ATCC 43895 was assessed by measuring the OD_600_nm every hour for 6 h following infection with the phage at MOI values of 0.0001, 0.001, 0.01, and 0.1 (Figure 1B). Within 6 h post-infection, a significant decrease in the number of host bacterial cells was observed at all tested MOIs, revealing an MOI-dependent inhibition of bacterial growth. Notably, even at the lowest MOI of 0.0001, the OD_600_ values of the phage-treated groups were significantly lower than those of the positive control, indicating the potential efficacy of the phage in controlling STEC at low titers.

### 3.4. Adsorption Rate and One-Step Growth Curve of STEC Phage vB_ESM-pEJ01

To estimate the life cycle of phage vB_ESM-pEJ01, an adsorption assay and one-step growth curve analysis were conducted at an MOI of 0.01. The adsorption kinetics revealed that over 80% of free phage particles were adsorbed onto the host strain ATCC 43895 within 10 min, indicating efficient attachment of the phage to its host bacteria (Figure 1C). Based on adsorption affinity, the one-step growth curve of the phage demonstrated a latent period of 5 min and a burst size of 427 PFU/infected cells (Figure 1D).

### 3.5. Environmental Stability of STEC Phage vB_ESM-pEJ01

**Figure 2:**
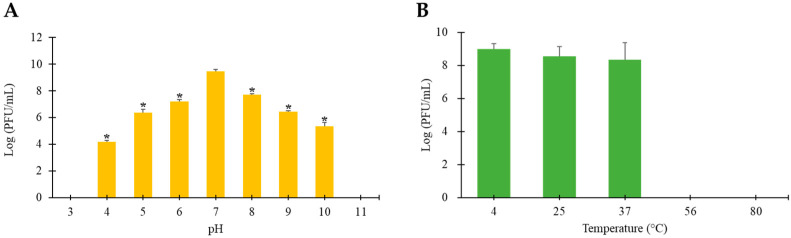
pH (**A**) and thermal (**B**) stability of STEC phage vB_ESM-pEJ01. The pH stability of the phage was determined at nine different pH values (pH 3–11) and the thermal stability of the phage was determined at five different temperatures (4–80 °C). Error bars represent the standard deviation of three replicates. Asterisks (*) indicate a statistically significant difference (*p* < 0.05).

To assess the stability of phage vB_ESM-pEJ01, pH- and thermal-stability tests were performed under various environmental conditions. The pH stability results revealed that the phage was relatively stable within a wide pH range of 4 to 10 but was completely inactivated under strongly acidic (pH 3) or alkaline (pH 11) conditions (Figure 2A). The thermal stability results observed that the phage was activated at temperatures ranging from 4 to 37 °C while significant inactivation was observed at temperatures above 45 °C (Figure 2B).

### 3.6. Anti-Biofilm Activity of STEC Phage vB_ESM-pEJ01

**Figure 3:**
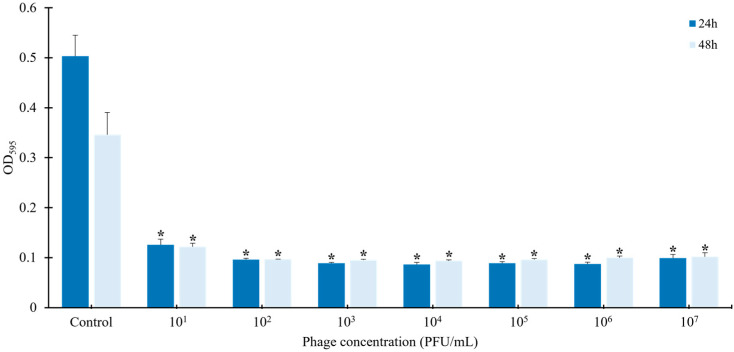
Anti-biofilm efficacy of STEC phage vB_ESM-pEJ01 on host bacterial strain ATCC 43895. The biofilm formation-inhibiting ability of phage was determined by measuring the OD595nm for the biofilm of host cells stained with crystal violet. Error bars represent the standard deviation of three replicates. Asterisks (*) indicate a statistically significant difference (*p* < 0.05).

To evaluate the effect of phage vB_ESM-pEJ01 on biofilm formation by the host strain ATCC 43895, bacterial cells were incubated with different concentrations of the phage suspension (10^1^, 10^2^, 10^3^, 10^4^, 10^5^, 10^6^, and 10^7^ PFU/mL) for 24 and 48 h (Figure 3). The results indicated that the phage effectively inhibited biofilm formation compared to the control group without phage treatment. Even at low phage concentrations, its anti-biofilm activity was consistently maintained across all tested phage titers, ranging from 10^1^ to 10^7^ PFU/mL, suggesting that the phage significantly reduced biofilm formation by STEC.

### 3.7. Genomic Characteristics of STEC Phage vB_ESM-pEJ01

**Figure 4:**
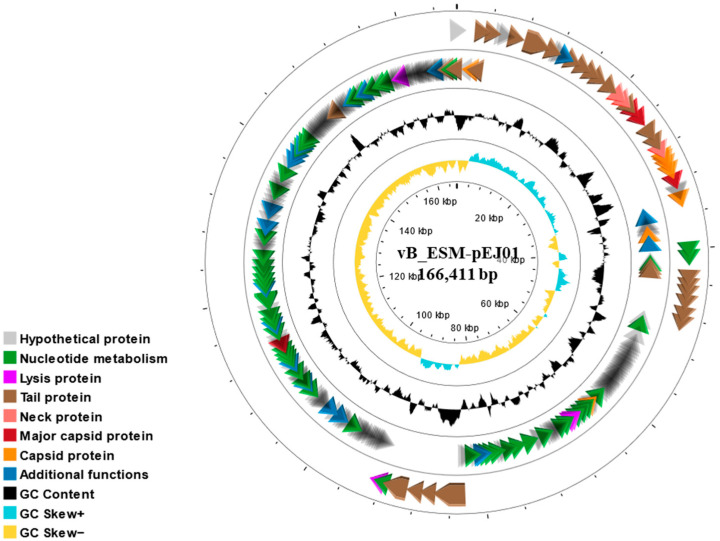
Circular genome map of STEC phage vB_ESM-pEJ01. The map is illustrated with the external and inner rings showing ten categories of genes encoding putative functional proteins and GC content with different colors using CGView.

The complete genome of STEC phage vB_ESM-pEJ01 was sequenced, revealing a linear double-stranded DNA of 166,422 bp with a GC content of 40.4%. Based on annotation analysis using RAST, 273 ORFs were identified (Figure 4). Among the ORFs, 114 were predicted to encode functional proteins, which were categorized into seven groups: nucleotide metabolism (47 ORFs), lysis proteins (four ORFs), tail proteins (29 ORFs), neck proteins (three ORFs), major capsid proteins (four ORFs), capsid proteins (eight ORF), and additional functional proteins (19 ORFs) (Supplementary Table S1). Most ORFs were similar to representative *Krischvirus* phages in the GenBank database, with amino acid identities ranging from 86.9 to 100%. Among these, four lysis-related genes were identified, encoding peptidoglycan hydrolase (ORF 6; amino acid identities, 98.5–99%), two phage spanins (ORF 89; amino acid identities, 95.3–98% and ORF 90; amino acid identities, 89.8–100%), holin (ORF 120; amino acid identities, 97.7–99.5%), and endolysin (ORF 248; amino acid identities, 91.3–100%), in *Krischvirus* phage genomes (Supplementary Table S2). No tRNAs, bacterial virulence- or antimicrobial resistance-associated genes, or lysogenicity-associated genes were detected in the genome. A BLASTn comparison revealed that the genome of phage vB_ESM-pEJ01 exhibited the highest similarity to previously reported *Krischvirus* phages: Enterobacteria phage RB49 (97.2% identity and 95% coverage; NC_005066.1) and *Escherichia* phage KFS-EC (96.9% identity and 93% coverage; NC_055757.1).

### 3.8. Phylogenetic and Genomic Comparison Analysis of STEC Phage vB_ESM-pEJ01

**Figure 5:**
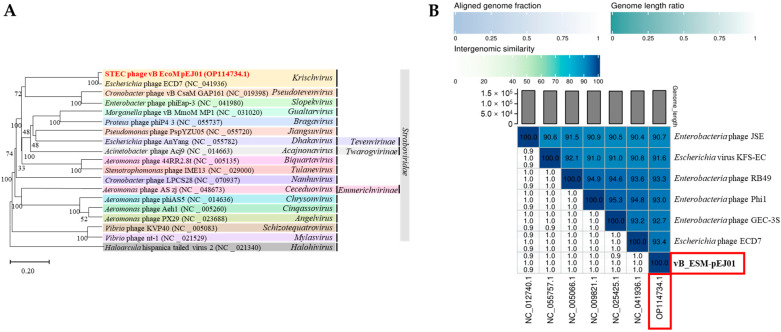
(**A**) VICOTR-based Genome BLAST Distance Phylogeny (GBDP) tree of STEC phage vB_ESM-pEJ01. A tree was constructed with the 19 other phages belonging to the *Straboviridae* family. (**B**) A VIRIDIC heat map showing the intergenomic similarity between the phage (red box) and six *Krischvirus* phages. The right side of the heatmap uses a gradient of blue hues to represent the similarity percentage among the genomes, with darker shades indicating higher similarity. The left side displays three genomic metrics: the aligned genome fraction for each genome in the rows (upper section), genome length ratios (middle section), and the aligned genome fraction for the genome in each column (lower section).

**Figure 6:**
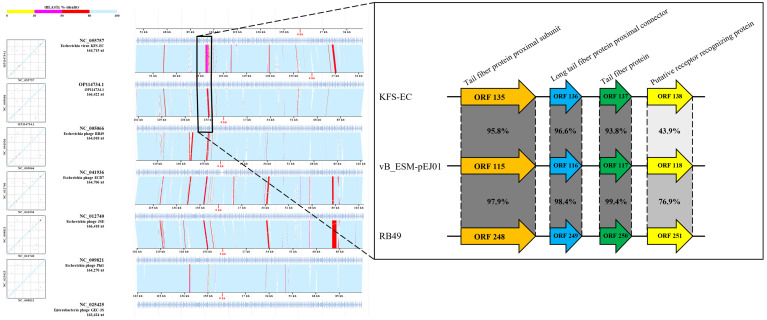
Genomic comparison between STEC phage vB_ESM-pEJ01 and six *Krischvirus* phages using ViPTree. The light blue arrows indicate the ORFs, while the colored lines connecting the genomes represent homologous regions detected by a tBLASTx search, with colors corresponding to amino acid identity (%) as shown in the color bar.

A VICTOR-based phylogenetic analysis using whole-genome sequences of the STEC phage vB_ESM-pEJ01 and 17 other related phages within the family *Straboviridae* revealed that the phage was phylogenetically more closely related to the genus *Krischvirus* (Figure 5A). In addition, based on the average nucleotide identity (ANI) values calculated using VIRIDIC, a heatmap was constructed with high intergenomic similarity (>90%) among STEC phages belonging to the genus *Krischvirus* available in the GenBank (Figure 5B). Additional phylogenetic trees were constructed using functional proteins, including major capsid protein (Supplementary Figure S1A) and terminase large subunit (Supplementary Figure S1B). These analyses demonstrated that phage vB_ESM-pEJ01 clustered into distinct branches separate from other *Krischvirus* phages, suggesting a unique taxonomic position within this genus. Genome alignment was performed using ViPTree to determine the most homologous regions in the genomes of the phage and six *Krischvirus* phages. Interestingly, a unique sequential cluster comprising four tail fiber proteins was identified (Figure 6), including tail fiber protein proximal subunit (ORF 115; amino acid identities, 95.7–99.2%), long tail fiber protein proximal connector (ORF 116; amino acid identities, 96.5–99.2%), tail fiber protein (ORF 117; amino acid identities, 93.7–99.6%), and large distal tail fiver subunit (ORF 118; amino acid identities, 43.8–78.1%), which is a characteristic feature of the T4-like coliphage subgroup RB49 genome (Supplementary Table S2).

### 3.9. Biocontrol Efficacy of STEC Phage vB_ESM-pEJ01 Against STEC-Contaminated Green Juice

**Figure 7:**
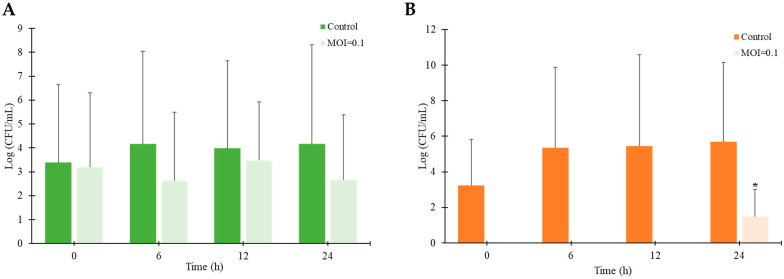
Efficacy of STEC phage vB_ESM-pEJ01 in STEC-contaminated green juice under the incubation at 4 (**A**) and 25 °C (**B**). The viable host cells were counted on STEC CHROMagar after phage treatment for 0, 6, 12, and 24 h compared to the control group without phage. The error bars represent the standard deviation of three replicates. Asterisks (*) indicate a statistically significant difference (*p* < 0.05).

**Figure 8:**
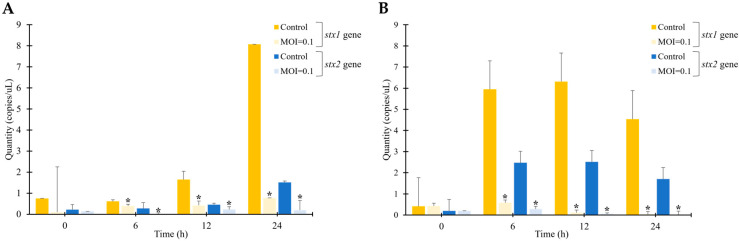
Absolute abundance of *stx*1 and *stx*2 genes of STEC phage vB_ESM-pEJ01 in STEC-contaminated green juice under the incubation at 4 (**A**) and 25 °C (**B**). Both the *stx* genes were quantified using a standard curve generated with qPCR, following phage treatment for 0, 6, 12, and 24 h compared to the control group without phage. The error bars represent the standard deviation of three replicates. Asterisks (*) indicate a statistically significant difference (*p* < 0.05).

To evaluate the efficacy of phage vB_ESM-pEJ01 in controlling STEC contamination in foods, green juice samples were inoculated with the host bacterial strain ATCC 43895 and incubated at 4 and 25 °C. The effectiveness of the phage was assessed by determining the viable host cell counts and quantifying the abundance of Shiga toxin-producing genes, including *stx*1 and *stx*2, in the incubated samples. After 24 h of incubation at 4 °C, the phage-treated group exhibited a significant reduction in viable host cell counts by 1.5 log CFU/mL compared to the control group (Figure 7A). After 12 h of incubation at 25 °C, the phage treatment demonstrated a significant decrease in host cell growth, with a 2.7 log CFU/mL reduction (Figure 7B). However, bacterial regrowth was observed after 24 h at 25 °C, indicating that the phage efficacy in controlling STEC was more maintained at the lower incubation temperature at 4 °C. After 24 h of incubation at 4 °C, the phage vB_ESM-pEJ01-treated group at an MOI of 0.1 showed a significant difference in the abundance of the *stx* genes compared to their respective control groups without phage treatment (Figure 8A). Under this condition, the quantity of the *stx*1 gene (0.78 copies/L) was higher than that of the *stx*2 gene (0.2 copies/L) in the phage-treated samples. At 25 °C, the absolute abundance of the representative *stx* genes exhibited a significant reduction over a 12 h incubation period (Figure 8B). These results demonstrated the efficacy of phage in controlling pathogenic bacteria in STEC-contaminated food under different storage conditions.

## 4. Discussion

The increasing prevalence of STEC infections with multidrug-resistant bacteria has gained significant interest as alternative antimicrobial agents, such as phages, to enhance food safety [10,11,13]. Recently, phage cocktails combining single species-specific phages, such as ELY-1 (*E*. *coli*) and phSE-5 (*S*. Typhimurium) [15], EC4 (*E*. *coli*) and φ135 (*Salmonella* Enteritidis) [43], and 153T 3ii (*E*. *coli*) and 191(3) (*Salmonella* Weltevreden) [44], have demonstrated efficacy in controlling pathogenic bacteria in food matrices. Although the unique features of host species-specific (or even strain-specific) phages are advantageous for biocontrol, their narrow host range can be a limitation. The use of polyvalent phages, which exhibit broader host ranges across multiple bacterial species and genera, can overcome the challenges posed by phage cocktails, including time-consuming and costly development, the risk of cross-resistance, and potential imbalances in microbial communities [16,21]. Several studies have demonstrated the potential of polyvalent phages to infect multiple bacterial species and genera. The identification of polyvalent phages, such as PS5 [19], LPEK22 [45], and EP01 [46], which target both *Salmonella* and *E. coli* strains, has shown promising applications, exhibiting efficacy both in vitro and in food products, including chicken, beef, and milk.

In this study, STEC phage vB_ESM-pEJ01 exhibited broad-spectrum activity. It demonstrated efficacy in controlling STEC contamination of green juice and belonging to the *Myoviridae* morphotype, phage vB_ESM-pEJ01 infected STEC strains, non-STEC strains, other *Escherichia* species (*E*. *hermanni* and *E*. *fergusonii*), and different serovars of *Salmonella* (*S.* Enteritidis, *S.* Typhimurium, *S.* Agona, *S.* Java, and *S.* Heidelberg). This cross-genera infectivity highlights the potential of the phage as a polyvalent phage against foodborne pathogens, potentially simplifying phage cocktail formulations. For the future scale-up of phage production, propagating phage vB_ESM-pEJ01 with appropriately selected non-STEC strains could minimize the acquisition of unwanted bacteria-mediated endotoxins or virulence-related genes, thereby ensuring the safety of phage applicability.

The bacteriolytic activity of phage vB_ESM-pEJ01 showed an effective MOI-dependent inhibitory activity throughout the incubation period. Remarkably, even at an ultra-low MOI of 0.0001, the phage exhibited potent inhibition of bacterial growth compared to other polyvalent phages such as vB_STM-2 [47] and Sa45lw [48], which required significantly higher MOIs of 100 and 10, respectively, to achieve bacterial reduction. The ability of the phage to effectively control foodborne pathogens at low concentrations is highly desirable, as it makes phage vB_ESM-pEJ01 an efficient and economical biocontrol agent for various food industry applications during food processing and preservation. One-step growth curve analysis revealed that phage vB_ESM-pEJ01 had a significantly shorter latent period of 5 min than other reported polyvalent phages, such as KFS-EC3 (20 min) [49] and Sa157lw (30 min) [48], indicating its ability to rapidly replicate and lyse host bacteria within a short time. Furthermore, the phage exhibited a considerably larger burst size of 427 PFU/infected cells than the representative polyvalent phages, KFS-EC3 (71 PFU/infected cells) [49] and Sa157lw (130 PFU/infected cells) [48]. This property supports the ability to effectively propagate and infect a large number of host bacteria in a given time. The lytic cycle characteristics of phage vB_ESM-pEJ01 are crucial for determining the efficacy of phage-based biocontrol strategies and suggest its potential as an effective biocontrol agent. Moreover, the phage was stable over a wide pH range (pH 4–10), similar to that of the polyvalent *Salmonella* phage Sa45lw (pH 4–10) [48]. Additionally, the phage maintained its infectivity up to 37 °C; however, its thermal stability was lower than that of some polyvalent phages, such as Sa45lw and KFS-EC3, which can withstand temperatures up to 60 °C [48,49], suggesting its potential use in combination with moderate heat treatments. The environmental stability of the phage at a wide pH range and moderate temperatures is advantageous for maintaining infectivity during processing and storage [10,12], potentially extending its shelf life [50], and making it well-suited for application in food-producing environments.

The anti-biofilm efficacy of phage vB_ESM-pEJ01 was demonstrated by its ability to inhibit STEC biofilm formation and significantly reduce its biomass after 24 and 48 h of treatment, even at low 10^1^ PFU/mL concentrations. This makes it a promising candidate for controlling STEC contamination, particularly in the food industry, where biofilm formation poses a significant challenge [51]. As a polyvalent phage targeting multiple species or genera, phage vB_ESM-pEJ01 can potentially prevent the growth of multiple pathogens; however, further studies are needed to investigate its efficacy against biofilms formed by other foodborne pathogens.

According to genome-based phylogenetic analyses, the STEC phage vB_ESM-pEJ01 belongs to the family *Straboviridae*, which includes well-studied T4-like phages (formerly T-even phages) known for their broad host spectrum and is divided into four significant subgroups (T4, RB69, RB49, and JS98) [52]. Specifically, phage vB_ESM-pEJ01 exhibited high nucleotide identity (>95%) with the RB49 subgroup (genus *Krischvirus*), which is characterized by a T4-like genome organization [53,54]. However, the phage possesses unique host-specificity-related genes, particularly tail fiber proteins, which distinguish it from other members of the RB49 subgroup. The phage genome encodes three tail fiber proteins (ORF 115–117) and a tail spike protein (ORF 118), which are considered host receptors. The unique composition of these tail-associated proteins is presumed to be the basis for their distinct polyvalent host range, similar to that of RB49 and KFS-EC phages, which are well-known to infect both *E*. *coli* and *Salmonella* [49,54]. The primary difference between phage vB_ESM-pEJ01 and closely related phages was revealed in the tail spike protein (ORF 118), which is associated with recognizing receptor proteins on bacterial membranes. The amino acid sequence of ORF 118 in phage vB_ESM-pEJ01 showed relatively low identity with those encoded in RB49 (>77%) and KFS-EC (>44%) phages, suggesting that this protein may confer a unique specificity to phage vB_ESM-pEJ01. Tail spike proteins act as receptor-binding proteins with high specificity and affinity, determining the host range infection efficiency of phages [55]. Thus, the unique composition of the tail spike protein in phage vB_ESM-pEJ01 suggests that this phage may be polyvalent, enabling it to recognize multiple receptor types on bacterial surfaces and infect a broader range of hosts compared to monovalent phages, which recognize a single receptor type [55,56]. However, further studies are needed to investigate the phage receptors across different host bacteria, which currently limits the broad infection range of phage vB_ESM-pEJ01.

To determine the efficacy of phage vB_ESM-pEJ01 in STEC-contaminated food, the present study assessed the viable host cell counts (CFU) in response to phage treatment at different MOIs. It quantified the abundance of Shiga toxin-producing genes (*stx*1 and *stx*2) at various temperatures (4 and 25 °C) and time points (0, 6, 12, and 24 h). The effectiveness of phage vB_ESM-pEJ01 was significantly higher at 25 °C compared to 4 °C, indicating that the phage has an excellent bactericidal effect even at room temperature. However, bacterial regrowth was observed 24 h after phage treatment at 25 °C, which could be attributed to either (i) the emergence of phage-resistant strains under more favorable growth conditions (25 °C; abuse temperature for RTE foods) [6] or (ii) the ability of storage under refrigerated conditions (4 °C; general temperature for food storage) to prevent bacterial regrowth after phage treatment. Notably, even at a low MOI of 0.1, phage vB_ESM-pEJ01 effectively inhibited the growth of STEC at both 4 and 25 °C, indicating that phage particles suspended in liquid foods can diffuse more freely than those on solid surfaces [57].

In response to the observed bacterial regrowth 24 h after phage treatment at 25 °C, we investigated the emergence of phage-resistant mutants to address the common concern in phage therapy. Interestingly, our results revealed that certain phage-resistant colonies had absent detection levels of *stx* genes (Supplementary Figure S2), potentially indicating the emergence of mutants with reduced virulence. As previously described, this partial loss of virulence genes in phage-resistant isolates may be attributed to phage-induced attenuation of bacterial pathogenicity [58]. These findings suggest that phage vB_ESM-pEJ01 controls STEC and selects for less virulent strains, making it a promising biocontrol candidate.

In contrast, several commonly used antibiotics have been reported to increase STEC pathogenicity by inducing prophages within the bacterial genome, resulting in the release of Shiga toxin-producing genes [59,60,61]. The phage vB_ESM-pEJ01 exhibited a significant reduction in the abundance of these genes in STEC. This supports the potential use of phage vB_ESM-pEJ01 as an alternative to antibiotics for the biocontrol of STEC infections and as a prophylactic treatment against bacteria.

## 5. Conclusions

This study reports the detailed characteristics of the newly isolated STEC phage vB_ESM-pEJ01, a polyvalent phage with a broad host range that efficiently infects *E*. *coli* isolates (STEC and non-STEC), other *Escherichia* species, and several *Salmonella* serovars. Moreover, the effective anti-biofilm activity and stability of the phage over wide pH and moderate temperature ranges further support its potential application in food processing and storage environments. Genome analysis revealed that phage vB_ESM-pEJ01 belongs to the *Krischvirus* family and possesses unique tail fiber and spike proteins that potentially contribute to its extensive host specificity. Notably, even at the lowest MOI tested, phage vB_ESM-pEJ01 demonstrated high efficiency in reducing STEC contamination and Shiga toxin-producing genes in artificially inoculated green juice. Thus, this study suggests that phage vB_ESM-pEJ01 is a promising biocontrol agent against foodborne pathogens, offering a potential alternative to the increasing occurrence of foodborne outbreaks associated with RTE foods such as fresh juices.

## Figures and Tables

**Table 1:** Primer features used in this study for qPCR.

Genes	Sequences (5′ to 3′)	Annealing Temperature (°C)	Efficiency *	Amplicon Size (bp)
*stx 1*	F: CATTACAGACTATTTCATCAGGAGGTA	55	1.98	88
	R: TCGTTCAACAATAAGCCGTAGATTA			
*stx 2*	F: GCGGTTTTATTTGCATTAGC	55	1.91	75
	R: TCCCGTCAACCTTCACTGTA			

* Efficiency is calculated as [10-1/slope].

**Table 2:** The host range of STEC phage vB_ESM-pEJ01.

Bacterial Species	Strains *	Sensitivity of Phage vB_ESM-pEJ01
*Escherichia* spp.		
Shiga toxin-producing *Escherichia coli* (STEC)	ATCC 43895	+
	KCCM 90572	+
	KCCM 90573	+
	KCCM 90574	+
	KCCM 90575	+
	KCCM 90576	+
	KCCM 90577	+
	KCCM 90578	+
	KCCM 90579	+
	KCCM 90580	+
	KCCM 90581	+
	KCCM 90582	+
	KCCM 90583	+
	KCCM 90584	−
	KCCM 90585	+
Non-STEC	ATCC 13706	+
	ATCC 11775	+
	ATCC 31616	+
	ATCC 31618	+
	ATCC 23545	+
	KCCM 90525	+
	KCCM 90526	+
Enterohemorrhagic *Escherichia coli* (EHEC)	NCCP 13720	+
	NCCP 13721	+
*Escherichia hermanni*	ATCC 33650	+
*Escherichia fergusonii*	ATCC 35469	+
*Salmonella* spp.		
*Salmonella* (*S.*) *enterica* serovar Enteritidis	KCTC 82777	+
	KCTC 82776	+
	KCCM 90531	+
	KCCM 90532	−
	KCCM 90533	−
	KCCM 90534	−
	KCCM 90535	+
	KCCM 90536	−
	KCCM 90537	−
	KCCM 90538	−
	KCCM 90539	−
	KCCM 90540	+
	KCCM 90541	−
	KCCM 90542	−
	KCCM 90543	−
	KCCM 90544	−
	KCCM 90545	−
	KCCM 90546	+
	KCCM 90547	-
	KCCM 90548	+
	KCCM 90549	−
	KCCM 90550	−
	KCCM 90551	−
	KCCM 90552	−
	KCCM 90553	+
	KCCM 90554	+
	KCCM 90555	+
	KCCM 90556	−
*S*. *enterica* serovar Typhimurium	KCCM 90557	−
	KCCM 90558	+
	KCCM 90559	+
	KCCM 90560	+
	KCCM 90561	+
	KCCM 90562	−
	KCCM 90563	−
	KCCM 90564	+
	KCCM 90565	+
	KCCM 90566	+
*S*. *enterica* serovar Agona	KCCM 90567	+
*S*. *enterica* serovar Bareilly	KCCM 90568	−
*S*. *enterica* serovar Infantis	KCCM 90569	−
*S*. *enterica* serovar Senftenberg	KCCM 90571	−
*S*. *enterica* serovar Motevideo	KCCM 90570	−
*S*. *enterica* serovar Java	NCTC 9683	+
*S*. *enterica* serovar Heidelberg	NCTC 14765	+

* KCCM, Korean Culture Center of Microorganisms; NCCP, National Culture Collection for Pathogens; ATCC, American Type Culture Collection; NCTC, National Collection of Type Cultures. +, complete lysis; −, no lysis.

## Data Availability

Data are contained within the article and Supplementary Materials. The STEC phage vB_ESM-pEJ01 was deposited in the Korean Collection for Type Cultures (KCTC) under KCTC 15809BP. The complete genome sequence of STEC phage vB_ESM-pEJ01 was deposited in the GenBank database of the National Center for Biotechnology Information (NCBI) under accession number OP114734.

## Supplementary Materials

The following supporting information can be downloaded at https://www.mdpi.com/article/10.3390/microorganisms13010103/s1, Supplementary Figure S1: Gene-phylogeny of STEC phage vB_ESM-pEJ01 based on the two genes encoding terminase large subunit (A) and major capsid protein (B); Supplementary Figure S2: (A) STEC Chrom agar plate showing the phage-resistant *E. coli* colonies obtained from the co-culture of the phage vB_ESM-pEJ01 and *E. coli* ATCC 43895. Colony PCR results showing the presence/absence of *stx1* (180 bp) (B) and *stx2* (255 bp) (C) genes in the representative phage-resistant *E. coli* colonies. The presence of the Shiga-toxin genes (*stx1* and *stx2*) in the phage-resistant *E*. *coli* colonies was confirmed using the previously reported PCR primers; Supplementary Table S1: Features of the predicted ORFs and their homology to the STEC phage vB_ESM-pEJ01.; Supplementary Table S2: Comparison of amino acid identities between STEC phage vB_ESM-pEJ01 and representative *Krischvirus* phages based on three functional ORF groups in the genome. Ref. [62] can be found in Supplementary Materials.
