# Supplementary material for: Characterization of Broad Spectrum Bacteriophage vB ESM-pEJ01 and Its Antimicrobial Efficacy Against Shiga Toxin-Producing Escherichia coli in Green Juice
Source: Microorganisms. 2025 Jan 7;13(1):103. doi: 10.3390/microorganisms13010103 (PMC11767321; doi:10.3390/microorganisms13010103)
Supplement: Supplementary file 1 [file microorganisms-13-00103-s001.zip › Supplementary Figure S1.pdf]

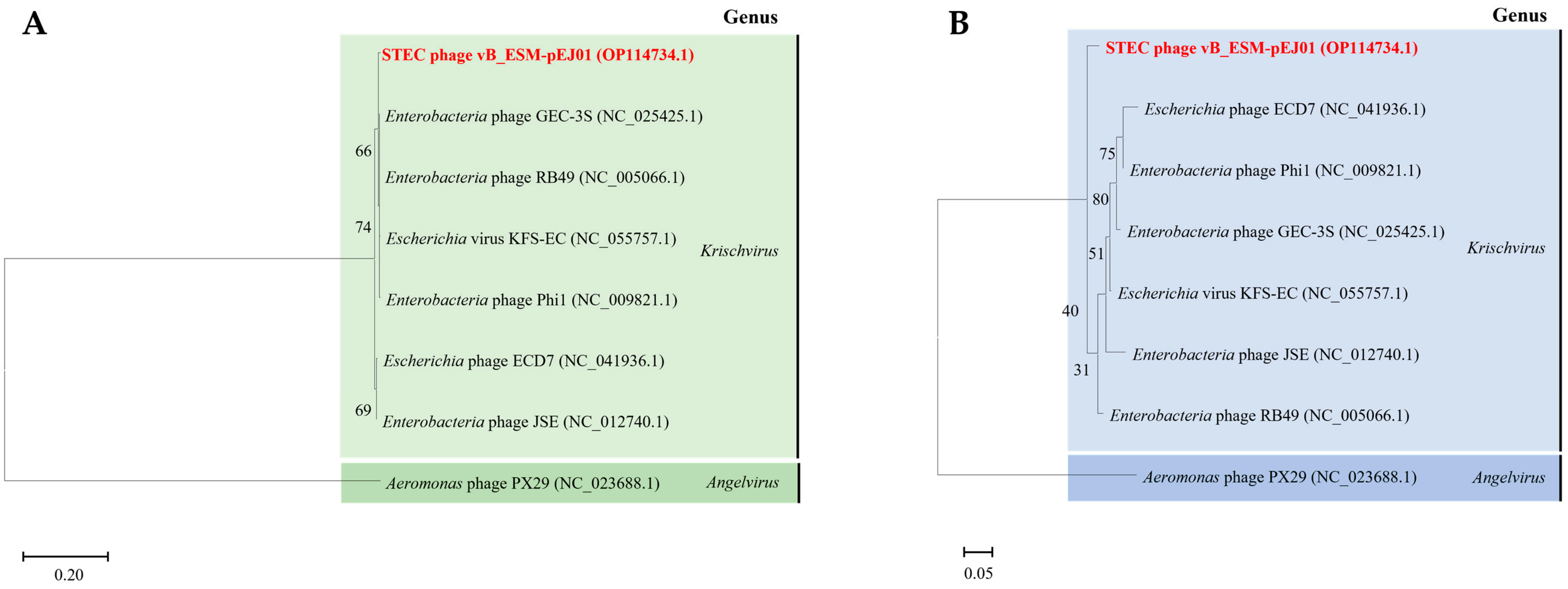

Supplementary Figure S1. Gene-phylogeny of STEC phage vB_ESM-pEJ01 based on the two genes encoding terminase large subunit (A) and major capsid protein (B). Two trees were constructed with six *Krischvirus* using the maximum-likelihood method by MEGA X. The bootstrap values supporting the branch are calculated with 1000 replicates.
