# Supplementary material for: Characterization of Broad Spectrum Bacteriophage vB ESM-pEJ01 and Its Antimicrobial Efficacy Against Shiga Toxin-Producing Escherichia coli in Green Juice
Source: Microorganisms. 2025 Jan 7;13(1):103. doi: 10.3390/microorganisms13010103 (PMC11767321; doi:10.3390/microorganisms13010103)
Supplement: Supplementary file 1 [file microorganisms-13-00103-s001.zip › Supplementary Figure S2(kim)-1.pdf]

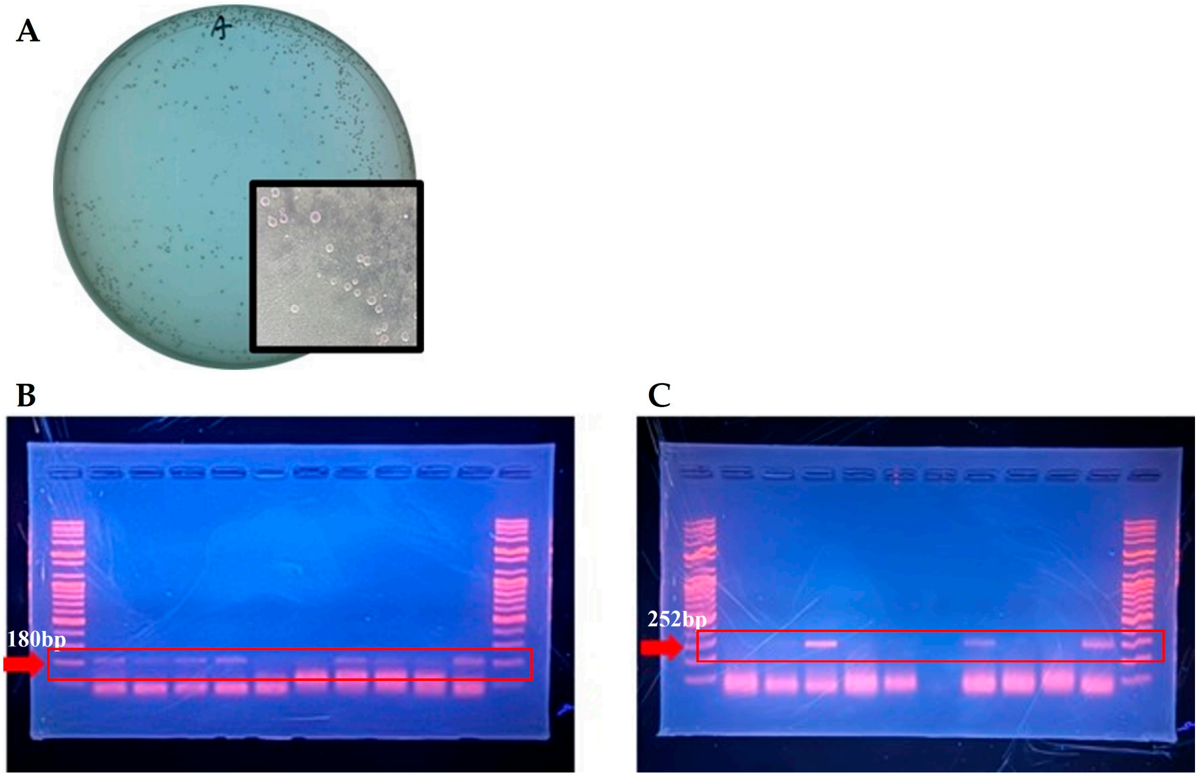

Supplementary Figure S2. (A) STEC Chrom agar plate showing the phage-resistant *E. coli* colonies obtained from the co-culture of the phage vB_ESM-pEJ01 and *E. coli* ATCC 43895. Colony PCR results showing the presence/absence of *stx1* (180 bp) (B) and *stx2* (255 bp) (C) genes in the representative phage-resistant *E. coli* colonies. The presence of the Shiga-toxin genes (*stx1* and *stx2*) in the phage-resistant *E. coli* colonies was confirmed using the previously reported PCR primers (Paton and Paton, 1998).

[Reference]

Paton, A. W., & Paton, J. C. Detection and characterization of Shiga toxigenic Escherichia coli by using multiplex PCR assays for *stx* 1, *stx* 2, *eaeA*, enterohemorrhagic *E. coli hlyA*, rfb *O111*, and rfb *O157*. Journal of Clinical Microbiology, **1998**, 36(2), 598-602. https://doi.org/10.1128/jcm.36.2.598-602.1998
