# Supplementary material for: Characterization of Broad Spectrum Bacteriophage vB ESM-pEJ01 and Its Antimicrobial Efficacy Against Shiga Toxin-Producing Escherichia coli in Green Juice
Source: Microorganisms. 2025 Jan 7;13(1):103. doi: 10.3390/microorganisms13010103 (PMC11767321; doi:10.3390/microorganisms13010103)
Supplement: Supplementary file 1 [file microorganisms-13-00103-s001.zip › Supplementary Table S1.pdf]

Supplementary Table S1. Features of the predicted ORFs and their homology to the STEC phage vB_ESM-pEJ01.

Gene product				Putative function [Conserved domain]	Best match organism (E-value)	Identit y (%)	Predicted TMH and signal peptide	
ORF No.	Range	Strand	Length (AA)				TMHHM	SignalP
1	37-291	+	84	Hypothetical protein	*Escherichia* phage vB_EcoM_PHB13(7e-51)	98.8	0	N
2	323-1351	-	342	Endonuclease	*Escherichia* phage ECD7 (0.0)	98.5	0	N
3	1344-2165	-	273	DNA end protector protein	*Escherichia* phage ECD7 (0.0)	99.6	0	N
4	2177-2650	-	157	Head closure<br>[MF_04160; IPR046390; NUCL_HEAD_T4; Head completion nuclease]	*Escherichia* phage Phi (9e-112)	99.4	0	N
5	2706-3260	+	184	Baseplate wedge subunit<br>[PF11246; IPR022607; Phage_T4_Gp53_baseplate_wedge; Baseplate wedge protein gp53, bacteriophage T4]	*Escherichia* phage RB49 (4e-134)	100	0	N
6	3272-5071	+	599	Baseplate hub subunit and tail lysozyme<br>[MF_04151; IPR046397; NEEDLE_T4; Pre-baseplate central spike protein Gp5]	*Escherichia* phage Phi1 (0.0)	99	0	N
7	5080-5646	+	188	Hypothetical protein	*Escherichia* phage Phi1 (2e-133)	98.9	0	N
8	5646-5939	+	97	Hypothetical protein<br>[PF05488; IPR008727; PAAR_motif; PAAR motif]	*Escherichia phage vB_EcoM_G2248* (2e-62)	99	0	N
9	5936-7840	+	634	Baseplate wedge subunit<br>[MF_04102; IPR034698; GP6_T4; Baseplate wedge protein gp6]	*Escherichia* phage Phi1 (0.0)	99.8	0	N
10	7837-10923	+	1028	Baseplate wedge subunit<br>[MF_04103; IPR034697; GP7_T4; Baseplate wedge protein gp7]	*Escherichia* phage ECD7 (0.0)	99.1	1	N
11	10916-11911	+	331	Baseplate wedge subunit<br>[PF09215; IPR015298; Phage_T4_Gp8; Bacteriophage T4, Gp8]	*Escherichia* phage Phi1 (0.0)	100	0	N
12	12021-12278	+	85	Hypothetical protein	*Enterobacteria* phage GEC-3S (7e-57)	98.8	0	N
13	12266-12598	+	110	Hypothetical protein	*Escherichia* phage JSE (3e-77)	99.1	0	N
14	12598-13437	+	279	DNA methyltransferase<br>[PF05175; IPR007848; Small_mtfrase_dom; Methyltransferase small domain]	*Escherichia* phage ECD7 (0.0)	98.2	0	N
15	13513-14367	+	284	Baseplate wedge tail fiber protein connector<br>[PF07880; IPR008987; Baseplate_struct_prot_Gp9/10; Baseplate structural protein Gp9/Gp10]	*Escherichia* phage JSE (0.0)	99.7	0	N
16	14364-16166	+	600	Baseplate wedge subunit<br>[PF07880; IPR008987; *Baseplate_struct_prot_Gp9/10*; Baseplate structural protein Gp9/Gp10]	*Enterobacteria* phage GEC-3S (0.0)	99.5	0	N
17	16168-16812	+	214	Baseplate wedge subunit<br>[PF08677; IPR014791; Baseplate_struct_Gp11; Baseplate structural protein Gp11]	*Enterobacteria phage GEC-3S*(2e-152)	98.1	0	Y
18	16824-	+	466	Tail collar fiber protein	*Escherichia* phage ECD7 (0.0)	99.8	0	N

	18224			[PF07484; IPR011083; Phage_tail_collar_dom; Phage tail collar domain]				
19	18224-19993	+	589	Fibritin neck whisker<br>[PF07921; IPR012473; Fibritin_C; Fibritin C-terminal]	*Escherichia* phage RB49 (0.0)	98.1	0	N
20	20040-20972	+	310	Head-tail adaptor	*Escherichia virus* KFS-EC (0.0)	99	0	N
21	21009-21749	+	246	Head closure Hc2<br>[PF11649; IPR021674; Phage_T4_Gp14_neck-protein; Bacteriophage T4, Gp14, neck protein]	*Escherichia* phage Phi1 (1e-175)	97.2	0	N
22	21776-22609	+	277	Tail sheath stabilizer<br>[PF16724; IPR031997; T4-gp15_tss; Myoviridae tail sheath stabiliser]	*Escherichia* phage RB49 (0.0)	99.6	0	N
23	22623-23120	+	165	Terminase small subunit<br>[PF11053; IPR020342; Phage_T4_Gp16_DNA-pack; Bacteriophage T4, Gp16, DNA-packaging]	*Escherichia* phage RB49 (2e-117)	100	0	N
24	23117-24940	+	607	Terminase large subunit<br>[MF_04146; IPR044267;<br>Terminase_large_su_gp17-like; Terminase, large subunit, gp17-like]	*Escherichia* phage RB49 (0.0)	99.3	0	N
25	24975-26972	+	665	Tail sheath<br>[PF04984; IPR035089; Phage_sheath_subtilisin; Tail sheath protein, subtilisin-like domain]	*Escherichia* phage JSE (0.0)	91.8	0	N
26	27046-27540	+	164	Tail tube protein<br>[PF06841; IPR010667; Phage_T4_gp19; Bacterop[jage T4, Gp19, tail tube]	*Escherichia* phage JSE (2e-116)	98.2	0	N
27	27599-29164	+	521	Portal protein<br>[PF07230; IPR010823; Portal_Gp20; Portal protein Gp20]	*Escherichia* phage RB49 (0.0)	98.7	0	N
28	29165-29383	+	72	Prohead<br>[PF17634; IPR035114; GP67; Gene product 67]	*Enterobacteria* phage GEC-3S (2e-23)	98	0	N
29	29396-29803	+	135	Head scaffolding protein<br>[PIRSF004377; IPR016415; Phage_T4_Gp68; Bacteriophage T4, Rohead assembly protein gp68]	*Enterobacteria* phage vB_EcoM_G2248 (4e-89)	98.5	0	N
30	29815-30510	+	231	Prohead core scaffold protein and protease<br>[PF03420; IPR005082; Peptidase_U9_T4_prohead; Peptidase U9, T4 prohead protease]	*Escherichia* phage Phi1 (5e-167)	99.1	0	N
31	30542-31336	+	264	Head scaffolding protein	*Escherichia* phage ECD7 (0.0)	99.6	0	N
32	31396-32982	+	528	Major head protein<br>[MF_04117; IPR038997; CAPSID_Myoviridae; Major capsid protein, Myoviridae]	*Escherichia* phage RB49 (0.0)	95.3	0	N
33	33312-33485	+	57	Hypothetical protein	*Escherichia* phage Phi1 (2e-22)	93.3	0	N
34	33482-33634	+	50	Hypothetical protein	*Escherichia* phage Phi1 (2e-25)	98	0	N
35	33719-34960	+	413	Head vertex protein<br>[PF07068; IPR010762; Gp23/Gp24_T4-like; Capsid protein, T4-like bacteriophage-	*Escherichia* phage Phi1 (0.0)	99	0	N

				like]				
36	34961-35548	-	195	DprA-like DNA recombination-mediator protein [SSF102405; MCP/YpsA-like]	*Escherichia* phage RB49 (5e-142)	99	0	N
37	35963-36400	-	145	Hypothetical protein	*Escherichia* phage Phi1 (7e-101)	99.3	0	N
38	36447-36722	-	91	Hypothetical protein [PF11242; IPR021404; Phage_T4_Gp24.3; Bacteriophage T4, Gp24.3]	*Escherichia* phage JSE (6e-58)	97.8	0	N
39	36722-36919	-	65	Hypothetical protein	*Escherichia* phage JSE (2e-36)	96.9	2	N
40	37121-38335	-	404	Hoc-like head decoration [SM00409; IPR003599; Ig_sub; Immunoglobulin subtype]	Enterobacteria phage GEC-3S (0.0)	96.5	0	N
41	38601-39326	-	241	Minor head protein inhibitor of protease	*Escherichia* phage GEC-3S (1e-171)	97.9	0	N
42	39397-40899	+	500	DNA helicase [PF04851; IPR006935; Helicase/UvrB_N; Helicase/Uvrb, N-terminal]	*Escherichia* phage Phi1 (0.0)	98.8	0	N
43	40943-41179	+	78	DNA helicase [PF11637; IPR020975; UvsW-1_dom; UvsW.1 domain]	*Escherichia* phage RB49 (3e-47)	98.7	0	N
44	41243-41419	-	58	Hypothetical protein [PF10886; IPR024362; DUF2685; Protein of unknown function DUF2685]	*Escherichia* phage RB49 (3e-33)	100	0	N
45	41441-41848	-	135	Recombination repair and ssDNA binding protein [PF11056; IPR021289; UvsY; Recombination, repair and ssDNA binding protein UvsY]	*Escherichia* phage kvi (8e-92)	96.3	0	N
46	41896-42282	-	128	Baseplate wedge subunit [PF04965; IPR007048; IraD/Gp25-like; IraD/Gp25-like]	*Escherichia* phage moskry (3e-51)	58.3	0	N
47	42291-42920	-	209	Baseplate hub subunit [PF12322; IPR024364; T4_baseplate; Baseplate_phage_T4-like_Baseplate hub assembly protein, bacteriophage T4-like]	*Enterobacteria* phage GEC-3S (5e-151)	99.5	0	N
48	42991-43761	+	256	Baseplate hub assembly catalyst [PF12322; IPR024364; Baseplate_phage_T4-like; Baseplate hub assembly protein, bacteriophage T4-like]	*Escherichia* phage Phi1 (0.0)	99.2	0	N
49	43739-44848	+	369	Baseplate hub [PF09097; IPR015181; Phage_T4_Gp27_N; Bacteriophage T4, Gp27, baseplate hub, N-terminal]	*Escherichia* phage RB49 (0.0)	99.5	0	N
50	44848-45366	+	172	Baseplate hub distal subunit [PF11110; IPR024342; Phage_T4_Gp28; Bacteriophage baseplate structural protein Gp28]	*Enterobacteria* phage GEC-3S (1e-122)	99.4	0	N
51	45363-47096	+	577	Baseplate hub subunit and tail length	*Enterobacteria* phage GEC-3S (0.0)	99	1	N
52	47096-48154	+	352	Baseplate tail tube cap [PF11091; IPR024389; Gp48_T4-like; Baseplate tail-tube protein gp48, T4- like virus]	*Escherichia* phage Phi1 (0.0)	99.7	0	N
53	48154-	+	310	Tail tube	*Escherichia* phage ECD7 (0.0)	100	0	N

	49086			[PF06841; IPR010667; Phage_T4_gp19; Bacteriophage T4, Gp19, tail tube]				
54	49118-49603	-	161	Hypothetical protein [PS51257; PROKAR_LIPOPROTEIN; Prokaryotic membrane lipoprotein lipid attachment site profile]	*Escherichia* phage RB49 (2e-109)	98.1	0	Y
55	49600-50424	-	274	ADP-ribosyltransferase [PF03496; IPR003540; ADP-ribosyltransferase; ADP ribosyltransferase]	*Escherichia* phage JSE (0.0)	98.9	0	N
56	50503-52554	-	683	Alt-like RNA polymerase ADP-ribosyltransferase [MF_04139; IPR016225; Phage_T4_Alt-like; NAD—protein ADP-ribosyltransferase Alt-like]	*Escherichia* phage RB49 (0.0)	99.3	0	N
57	52622-52834	-	70	Hypothetical protein	*Escherichia* phage JSE (8e-41)	100	0	N
58	52908-53426	-	172	Hypothetical protein [PF03235; IPR004919; DUF262; Domain of unknown function DUF262]	*Enterobacteria* phage GEC-3S (3e-123)	98.3	0	N
59	53428-53757	-	109	Hypothetical protein	*Escherichia* phage RB49 (9e-74)	97.3	0	N
60	53757-54473	-	238	DNA methyltransferase [SSF53335; IPR029063; SAM-dependent_MTases_sf; S-adenosyl-L-methionine-dependent methyltransferase superfamilly]	*Escherichia* phage RB49 (9e-175)	97.9	0	N
61	54470-54721	-	83	Hypothetical protein	*Escherichia* phage ECD7 (2e-52)	98.8	0	N
62	54805-54972	-	55	Hypothetical protein	*Escherichia* phage RB49 (2e-31)	98.2	0	N
63	55049-55330	-	93	Hypothetical protein	*Escherichia* phage RB49 (6e-61)	100	0	N
64	55401-55670	-	89	Hypothetical protein	*Escherichia* phage ECD7 (1e-59)	100	0	N
65	55796-56104	-	102	Hypothetical protein	*Escherichia* phage Phi1 (4e-65)	94.1	0	N
66	56154-56441	-	95	Hypothetical protein	*Enterobacteria* phage GEC-3S (4e-62)	99	0	N
67	56523-56801	-	92	Hypothetical protein [PF08084; PROCT; PROCT (NUC072) domain]	*Escherichia* phage RB49 (6e-60)	97.8	0	N
68	56816-57124	-	102	Hypothetical protein	*Escherichia* phage RB49 (9e-66)	99	0	N
69	57126-57356	-	76	Hypothetical protein	*Escherichia* virus KFS-EC(9e-49)	100	0	N
70	57548-57826	-	92	Hypothetical protein	*Escherichia* virus KFS-EC(1e-61)	100	0	N
71	57839-58018	-	59	Hypothetical protein	*Escherichia* virus KFS-EC(3e-35)	100	0	N
72	58083-	-	55	Hypothetical protein	*Escherichia* phage ECD7 (3e-31)	100	0	N

	58250							
73	58321-58623	-	100	Hypothetical protein	*Enterobacteria* phage GEC-3S (1e-64)	96	0	N
74	58640-58828	-	62	Hypothetical protein	*Escherichia* phage Phi1 (1e-36)	98.4	0	N
75	58885-59187	-	100	Hypothetical protein	*Escherichia* phage vB_EcoM_G2248 (2e-66)	100	0	N
76	59189-59455	-	88	Hypothetical protein	*Escherichia* phage JSE (1e-57)	100	0	N
77	59475-59654	-	59	Hypothetical protein	*Escherichia* virus KFS-EC (5e-3)	98.3	0	N
78	59727-60038	-	103	Hypothetical protein	*Escherichia* phage Phi1 (1e-68)	100	0	N
79	60126-60602	-	158	Hypothetical protein	*Enterobacteria* phage GEC-3S (3e-114)	100	0	N
80	60595-62091	-	498	ATP-dependent DNA ligase [PF01068; IPR012310; DNA_ligase_ATP-dep_cent; DNA ligase, ATP-dependent, central]	*Escherichia* virus KFS-EC (0.0)	99.8	0	N
81	62078-62557	-	159	Hypothetical protein [PF08010; IPR012596; Phage_T4_Y12G; Bacteriophage T4, Y12G]	*Escherichia* virus KFS-EC (2e-113)	98.7	0	N
82	62547-62711	-	54	Hypothetical protein	*Escherichia* phage RB49 (2e-29)	98.2	0	N
83	62795-63118	-	107	Head morphogenesis [PF00166; IPR020818; Chaperonin_GroES; GroES chaperonin family]	*Escherichia* phage ECD7 (2e-68)	100	0	N
84	63155-63766	-	203	HNH endonuclease [PF07463; IPR010902; NUMOD4; NUMOD4]	*Escherichia* phage ECD7 (3e-147)	99	0	N
85	63769-64089	-	106	Hypothetical protein [PF10902; IPR024401; WYL_prot; WYL_prot]	*Escherichia* virus KFS-EC (2e-69)	97.2	0	N
86	64082-64588	-	168	dCMP deaminase [PTHR11086; IPR015517; dCMP_deaminase-rel; Deoxycytidylate deaminase-related]	*Escherichia* virus KFS-EC (2e-120)	98.8	0	N
87	64610-64813	-	67	Hypothetical protein	*Escherichia* phage vB_EcoM_PHB13 (2e-41)	98.5	0	N
88	64813-65691	-	292	Polynucleotide kinase [G3DSA:3,40,50,300; IPR027417; P-loop_NTPase; P-loop containing nucleoside triphosphate hydrolase]	*Escherichia* phage Phi1 (0.0)	99.3	0	N
89	65691-66143	-	150	Rz-like spanin	*Escherichia* phage ECD7 (8e-105)	98	2	N
90	66140-66463	-	107	Rz-like spanin	*Enterobacteria* phage GEC-3S (1e-69)	98.1	1	Y

91	66508-67626	-	372	RNA ligase and tail fiber protein attachment catalyst [TIGR02308; IPR012648; Rnl1, T4 RNA ligase 1]	*Escherichia* virus KFS-EC (0.0)	99.5	0	N
92	67613-68074	-	153	Endonuclease [PIRSF004362; IPR016413; Phage_T4_denA_dendoDNase II; Bacteriophage T4, DenA, endonuclease II]	*Enterobacteria* phage GEC-3S (2e-107)	98.7	0	N
93	68125-68523	-	132	Hypothetical protein	*Escherichia* phage vB_EcoM_011D4 (2e-90)	99.2	0	N
94	68594-68743	-	49	Hypothetical protein	*Escherichia* phage vB_EcoM_011D4 (4e-25)	95.9	0	N
95	68813-69031	-	72	Hypothetical protein	*Escherichia* phage kvi (2e-45)	98.6	0	N
96	69101-69319	-	72	Hypothetical protein	*Enterobacteria* phage GEC-3S (3e-44)	98.6	0	N
97	69421-69897	-	158	Polynucleotide kinase [G3DSA:3,40,50,1000; IPR023214; HAD_sf; HAD superfamily]	*Enterobacteria* phage GEC-3S (1e-111)	99.4	0	N
98	69894-71081	-	395	Ribonucleoside diphosphate reductase small subunit [PF00268; IPR000358; RNR_small_fam; Ribonucleotide reductase small subunit family]	*Enterobacteria* phage GEC-3S (0.0)	97	0	N
99	71097-71264	-	55	Hypothetical protein	*Escherichia* phage RB49 (2e-31)	100	0	Y
100	71264-71437	-	57	Hypothetical protein	*Escherichia* virus KFS-EC (2e-33)	98.3	0	Y
101	71672-73915	-	747	NrdA-like aerobic NDP reductase large subunit [PTHR11573; IPR039718; Rrm1;Ribonucleoside-diphosphate reductase large subunit]	*Escherichia* virus KFS-EC (0.0)	99.9	0	N
102	73986-75206	-	406	Thymidylate synthase [MF_0008; IPR000398; Thymidylate_synthase; Thymidylate synthase]	*Escherichia* phage RB49 (0.0)	97.3	0	N
103	75541-76122	-	193	Dihydrofolate reductase [PTHR48069; IPR012259; DHFR; Dihydrofolate reductase]	*Escherichia* phage JSE (1e-136)	98.5	0	N
104	76138-76467	-	109	Hypothetical protein	*Escherichia* phage RB49 (2e-74)	99.1	0	N
105	76500-76937	-	145	Hypothetical protein [PF09669; IPR014054; Phage_regulate_Rha; Phage regulatory protein, Rha family]	*Vibrio* phage 184E37.3a (2e-15)	53.3	0	N
106	77054-78028	-	324	Single strand DNA binding protein [MF_04152; IPR046395; SSB_T4; Bacteriophage T4, Gp32, single-stranded DNA-binding]	*Escherichia* virus KFS-EC (0.0)	99.7	0	N
107	78089-78685	-	198	Putative HNH endonuclease [PF13392; IPR003615; HNH_nuc; HNH nuclease]	*Escherichia* phage Ec_Makalu_002 (1e-143)	99	0	N
108	78672-79349	-	225	DNA helicase loader [MF_04156; IPR008944; Phage_T4_Gp59; Bacteriophage T4, Gp59, helicase assembly protein]	*Escherichia* virus KFS-EC (9e-161)	98.7	0	N

109	79359-79613	-	84	Late promoter transcriptional regulator<br>[PF16805; IPR031836; Trans_coact; Late transcription coactivator]	*Escherichia* phage ECD7 (4e-53)	98.8	0	N
110	79606-79881	-	91	Transcriptional regulator<br>[PF11126; IPR020313; Double-stranded_DNA-bd; Double-stranded DNA-binding protein]	*Escherichia* phage RB49 (3e-58)	100	0	N
111	79886-80482	-	198	Hypothetical protein<br>[SM01118; IPR023577; CYTH_domain; CYTH domain]	*Escherichia* phage RB49 (2e-139)	97.5	0	N
112	80493-81440	-	315	RnaseH<br>[PTHR42646; IPR038969; FEN; Flap endonuclease]	*Escherichia* phage RB49 (0.0)	99.7	0	N
113	81443-81628	-	61	Hypothetical protein	*Escherichia* phage Phi1 (1e-34)	98.4	0	N
114	81635-82198	-	187	Hypothetical protein<br>[SM00380; IPR001471; AP2/ERF_dom; AP2/ERF domain]	*Escherichia* phage vB_EcoM_G37-3 (5e-137)	100	0	N
115	82251-85991	+	1246	Tail fiber protein proximal subunit<br>[cd19958; pyocin_knob; knob domain of R1 and R2 pyocins and similar domains]	*Escherichia* phage JSE (0.0)	98.4	0	N
116	86025-87164	+	379	Long tail fiber protein proximal connector	*Escherichia* phage Phi1 (0.0)	99.2	0	N
117	87367-88857	+	496	Tail fiber protein<br>[cd19958; pyocin_knob; knob domain of R1 and R2 pyocins and similar domains]	*Escherichia* phage Phi1 (0.0)	99.6	0	N
118	88928-91834	+	968	Large distal tail fiber subunit<br>[PS51688; IPR030392; S74_ICA; Intramolecular chaperone auto-processing domain]	*Escherichia* phage W115 (0.0)	85.8	0	N
119	91876-92115	+	79	Dc5	*Escherichia* phage RB49 (4e-39)	100	0	N
120	92149-92805	+	218	Holin<br>[MF_04104; IPR020982; Phage_T4_GpT_holin; Bacteriophage T4, GpT, holin]	*Escherichia* phage RB49 (5e-159)	99.5	0	N
121	92841-93089	-	82	Hypothetical protein	*Escherichia* phage vB_EcoM_G2248 (3e-54)	98.8	0	N
122	93086-93397	-	103	Hypothetical protein	*Escherichia* phage ECD7 (2e-68)	100	0	N
123	93422-93754	-	110	Hypothetical protein	*Escherichia* phage ECD7 (1e-74)	100	0	N
124	93763-94131	-	122	Hypothetical protein	*Escherichia* phage RB49 (2e-84)	99.2	0	N
125	94142-94333	-	63	Hypothetical protein	*Escherichia* phage ECD7 (4e-39)	100	0	N
126	94356-94643	-	95	Hypothetical protein	*Escherichia* phage RB49 (2e-62)	96.8	0	N
127	94655-95413	-	252	Hypothetical protein	*Escherichia* phage RB49 (0.0)	99.6	0	Y
128	95484-	-	70	Hypothetical protein	*Escherichia* phage RB49 (3e-40)	98.6	0	N

	95696							
129	95762-95923	-	53	Hypothetical protein	*Escherichia* phage Phi1 (5e-29)	100	0	N
130	95928-96200	-	90	Hypothetical protein	*Escherichia* virus KFS-EC (4e-59)	98.9	0	N
131	96267-96473	-	68	Hypothetical protein	*Escherichia* phage vB_EcoM_G37-3 (1e-41)	97.1	0	N
132	96587-96982	-	131	Hypothetical protein	*Escherichia* virus KFS-EC (6e-90)	98.5	0	N
133	96975-97181	-	68	Hypothetical protein	*Escherichia* phage Phi1 (5e-35)	96.8	0	N
134	97184-98530	-	448	DNA topoisomerase II [PF00521; IPR002205; Topo_IIA_dom_A]	*Escherichia* virus KFS-EC (0.0)	99.3	0	N
135	98538-98900	-	120	Hypothetical protein	*Escherichia* virus KFS-EC (2e-79)	99.2	2	N
136	98903-99196	-	97	Hypothetical protein	*Escherichia* phage RB49 (1e-61)	100	0	N
137	99242-99568	-	108	Hypothetical protein	*Escherichia* phage JSE (3e-71)	99.1	0	N
138	99583-100575	-	330	RIIB lysis inhibitor	*Escherichia* phage Phi1 (0.0)	98.8	0	N
139	100638-100769	-	43	Hypothetical protein [PF04218; IPR007889; HTH_Psq_DNA binding HTH domain, Psq-type]	*Escherichia* phage vB_EcoM_G5211 (6e-20)	97.7	0	N
140	100781-102889	-	702	RIIA lysis inhibitor [IPR036890; HATPase_C_sf; Histidine kinase/HSP90-like ATPase superfamily]	*Escherichia* phage RB49 (0.0)	99.4	0	N
141	102904-103161	-	85	Hypothetical protein	*Escherichia* phage JSE (1e-54)	97.7	0	N
142	103172-103366	-	64	Hypothetical protein	*Escherichia* phage JSE (2e-36)	98.4	0	N
143	103359-103535	-	58	Hypothetical protein	*Escherichia* phage RB49 (6e-27)	100	0	N
144	103618-103953	-	111	Hypothetical protein	*Escherichia* phage JSE (4e-66)	100	0	N
145	103976-104539	-	187	Hypothetical protein [SM00380; IPR001471; AP2/ERF_dom; AP2/ERF domain]	*Escherichia* phage JSE (2e-136)	98.9	0	N
146	104541-106364	-	607	DNA topoisomerase II large subunit [SM00433; IPR001241; Topo_IIA; DNA topoisomerase, type IIA]	*Escherichia* phage JSE (0.0)	99.7	0	N
147	106357-106467	-	36	Hypothetical protein	*Escherichia* phage Phi1 (7e-16)	100	1	N
148	106464-	-	57	FmdB-like transcriptional regulator	*Escherichia* phage RB49 (6e-34)	100	0	N

	106637			[SM00834; IPR013429; Regulatory_FmdB_Zinc_ribbon; Putative regulatory protein, FmdB, Zinc ribbon domain]				
149	106639-107307	-	222	Exonuclease<br>[PF16473; IPR033390; Rv2179c-like; 3'-5' exoribonuclease Rv2179c-like domain]	*Escherichia* phage Phi1 (e-165)	100	0	N
150	107309-107551	-	80	Hypothetical protein	*Escherichia* phage Phi1 (2e-49)	96.3	0	N
151	107639-107728	-	29	Hypothetical protein	*Escherichia* phage Phi1 (9e-10)	92.9	0	N
152	107730-107897	-	55	Hypothetical protein	*Escherichia* phage Phi1 (2e-33)	100	0	N
153	107908-109299	-	463	Exonuclease V<br>[SM00382; IPR003593; AAA+_ATPase; AAA+ ATPase domain]	*Escherichia* phage RB49 (0.0)	99.8	0	N
154	109299-109613	-	104	Hypothetical protein	*Escherichia* phage RB49 (1e-69)	100	0	N
155	109613-110368	-	251	Srd anti-sigma factor	*Escherichia* phage ECD7 (0.0)	99.2	0	N
156	110430-110933	-	167	ADP-ribosylase	*Escherichia*phagevB_EcoM_011D4 (9e-119)	99.4	0	N
157	111003-111521	-	172	dCTP pyrophosphatase	*Escherichia* phage RB49 (8e-126)	100	0	N
158	111536-112564	-	342	DNA primase<br>[MF_04157; IPR046392; PRIMASE_T4; DNA primase, bacteriophage T4]	*Escherichia* phage RB49 (0.0)	99.7	0	N
159	112574-112777	-	67	Hypothetical protein	*Enterobacteria* phage GEC-3S)	97	0	N
160	112774-112932	-	52	Hypothetical protein	*Escherichia* phage RB49 (4-21)	100	2	N
161	112925-113101	-	58	Hypothetical protein<br>[PF07068; IPR010762; Gp23; /GP24_T4-like; Capsid protein, T4-like bacteriophage-like]	*Escherichia* phage Phi1 (1e-30)	94.8	0	N
162	113098-113472	-	124	Hypothetical protein	Shigella phage JK32 (3e-85)	99.2	0	N
163	113503-114138	-	211	Hypothetical protein	*Escherichia* phage virus KFS-EC (5e-150)	97.2	0	N
164	114189-115601	-	470	Helicase<br>[MF_04155; IPR046393; Helic_T4; Bacteriophage T4 Dna-B-like replicative helicase]	*Escherichia* phage RB49 (0.0)	100	0	N
165	115598-115915	-	105	Head vertex assembly chaperone<br>[PF11113; IPR021049; Phage_T4_Gp40; Bacterophage T4, Gp40, head assembly]	*Escherichia* phage Phi1 (2e-68)	99.1	0	N
166	115957-116706	-	249	Homing endonuclease<br>[IPR003615; HNH_nuc; HNH nuclease]	*Escherichia* virus KFS-EC (0.0)	99.6	0	N
167	116758-	-	356	DNA repair protein	*Escherichia* virus KFS-EC (0.0)	99.7	0	N

	117828			[PTHR45900; IPR013765; DNA_recomb/repair_RecA; DNA recombination and repair protein RecA]				
168	117840-118064	-	74	Hypothetical protein	*Escherichia* virus KFS-EC (2e-35)	100	0	N
169	118090-120768	-	892	DNA polymerase [PF00136; IPR006172; DNA-dir_DNA_pol_B; DNA-directed DNA polymerase, family B]	*Enterobacteria* phage GEC-3S (0.0)	99.7	0	N
170	120851-121213	-	120	Translation repressor protein [PF01818; IPR002702; Translat_repress_RegA; Translation repressor RegA]	*Escherichia* phage ECD7 (2e-81)	99.2	0	N
171	121215-121793	-	192	Clamp loader of DNA polymerase [MF_04163; IPR031868; Phage_clamp_gp62; Sliding-clamp-loader small subunit gp62]	*Escherichia* phage Phi1 (8e-140)	99	0	N
172	121793-122767	-	324	Clamp loader DNA [MF_04162; IPR046388; T4_Clamp_Loader_L; Sliding-clamp-loader large subunit]	*Escherichia* phage RB49 (0.0)	100	0	N
173	122833-123519	-	228	DNA polymerase processivity factor [MF_04161; IPR046389; Sliding_clamp_T4; Sliding clamp]	*Escherichia* phage RB49 (3e-166)	99.6	0	N
174	123550-123927	-	125	Hypothetical protein	*Escherichia* phage ECD7 (4e-86)	99.2	0	N
175	123932-124309	-	125	RNA polymerase binding [PF10789; IPR019725; Phage_T4_P15K_Rpol-bd; Bacteriophage T4, P15K, RNA polymerase binding]	*Escherichia* phage RB49 (2e-84)	100	0	N
176	124340-124531	-	63	Hypothetical protein	*Escherichia* phage JSE (8e-39)	98.4	0	N
177	124557-126239	-	560	SbcC-like subunit of palindrome specific endonuclease [PF13476; IPR038729; Rad50/SbcC_AAA; Rad50/SbcC-type AAA domain]	*Escherichia* phage RB49 (0.0)	99.8	0	N
178	126220-126483	-	87	Hypothetical protein	*Escherichia* phage RB49 (2e-56)	100	0	N
179	126480-127052	-	190	Hypothetical protein	*Escherichia* phage JSE (2e-137)	99	0	N
180	127027-127347	-	106	Hypothetical protein	*Escherichia* phage Phi1 (1e-70)	99.1	0	N
181	127344-127592	-	82	Hypothetical protein	*Enterobacteria* phage GEC-3S (3e-53)	100	0	N
182	127647-128672	-	341	Exonuclease subunit 1 [PF00149; IPR004843; Calcineurin-like_PHP_ApaH; Calcineurin-like phosphoesterase domain, ApaH type]	*Escherichia* virus KFS-EC (0.0)	99.7	0	N
183	128763-129050	-	95	Hypothetical protein [PF10849; IPR022558; DUF2654; Protein of unknown function DUF2654]	*Escherichia* phage JSE (6e-61)	97.9	0	N
184	129052-129180	-	42	Hypothetical protein	*Enterobacteria* phage GEC-3S (2e-20)	97.6	0	N
185	129269-	-	177	Late sigma transcription factor	*Escherichia* phage JSE (2e-129)	100	0	N

	129802			[MF_04164; IPR046386; T4_sigma-like_factor; RNA polymerase sigma-like factor]				
186	129843-131312	-	489	Hypothetical protein [PF00270; IPR011545; DEAD/DEAH_box_helicase_dom; DEAD/DEAH box helicase domain]	*Escherichia* phage RB49 (0.0)	99.8	0	N
187	131312-131599	-	95	Conserved hypothetical protein	*Escherichia* virus KFS-EC (2e-62)	99	0	N
188	131621-131779	-	52	Hypothetical protein [SSF57938; IPR036410; HSP_DnaJ_Cys-rich_dom_sf; Heat shock protein DnaJ, cysteine-rich domain superfamily]	*Escherichia* phage Phi1 (4e-28)	100	1	N
189	131769-132038	-	89	Glutaredoxin [SSF52833; IPR036249; Thioredoxin-like_sf; Thioredoxin0like superfamily]	*Escherichia* phage RB49 (1e-57)	10	0	N
190	132035-132511	-	158	Hypothetical protein	*Escherichia* phage RB49 (1e-111)	100	0	N
191	132513-133337	-	274	Hypothetical protein	*Escherichia* virus KFS-EC (0.0)	99.6	0	N
192	133350-133817	-	155	Hypothetical protein	*Escherichia* phage JSE (1e-107)	100	0	N
193	133822-134349	-	175	Hypothetical protein	*Escherichia* phage JSE (6e-104)	86.9	0	N
194	134360-134863	-	167	Anaerobic ribonucleotide reductase small subunit [PIRSF000368; IPR012837; NrdG; Ribonucleoside-triphosphate reductase activating, anaerobic	*Escherichia* phage RB49 (9e-119)	99.4	0	N
195	134853-135005	-	50	Hypothetical protein [PF10123; Mu-like_Pro; Mu-like prophage I protein]	*Escherichia* phage RB49 (1e-26)	98	0	N
196	135081-135608	-	175	Hypothetical protein	*Enterobacteria* phage GEC-3S (9e-130)	100	0	N
197	135626-135955	-	109	Hypothetical protein	*Escherichia* phage ECD7 (1e-73)	99.1	0	N
198	135979-136173	-	64	Hypothetical protein	*Escherichia* phage Phi1 (3e-18)	93	0	N
199	136274-138136	-	620	Anaerobic ribonucleoside reductase large subunit [PF13597; IPR012833; NrdD; Ribonucleoside-triphosphate reductase, anaerobic]	*Escherichia* phage RB49 (0.0)	99.3	0	N
200	138181-138675	-	164	Hypothetical protein	*Enterobacteria* phage GEC-3S (1e-111)	97.6	0	N
201	138730-139908	-	392	GoF mRNA metabolism modulator	*Escherichia* virus KFS-EC (0.0)	98.2	0	N
202	139965-140531	-	188	GoF mRNA metabolism modulator	*Escherichia* phage RB49 (1e-130)	98.4	0	N
203	140546-140818	-	90	Hypothetical protein	*Enterobacteria* phage GEC-3S (2e-56)	93.3	0	N

204	140882-141355	-	157	Endonuclease<br>[PF02945; IPR004211; Endonuclease_7; Recombination endonuclease VII]	*Escherichia* phage Phi1 (2e-113)	99.4	0	N
205	141380-141661	-	93	Thioredoxin<br>[PF00462; IPR00219; Glutaredoxin; Glutaredoxin]	*Escherichia* phage ECD7 (2e-59)	98.9	0	N
206	141663-142418	-	251	Putative HNH homing endonuclease<br>[PS51032; IPR001471; AP2/ERF_dom; AP2/ERF domain]	*Enterobacteria* phage GEC-3S (0.0)	100	0	N
207	142396-143211	-	271	DNA methyltransferase<br>[TIGR00571; IPR012327; MeTrfase_D12; D12 class N6 adenine-specific DNA methyltransferase]	*Enterobacteria* phage GEC-3S (0.0)	98.5	0	N
208	143211-143642	-	143	Hypothetical protein	*Escherichia* phage Phi1 (2e-100)	98.6	0	N
209	143653-143946	-	97	Hypothetical protein	*Enterobacteria* phage GEC-3S (2e-63)	99	0	N
210	143939-144160	-	73	Hypothetical protein	*Escherichia* virus KFS-EC (4e-44)	97.3	0	N
211	144153-144446	-	97	Hypothetical protein	*Escherichia* virus KFS-EC (5e-59)	99	0	N
212	144461-144805	-	114	Hypothetical protein	*Escherichia* phage W115 (3e-74)	96.5	0	N
213	144840-145148	-	102	Hypothetical protein	*Escherichia* virus KFS-EC (3e-68)	98	2	N
214	145159-145437	-	92	Hypothetical protein	*Enterobacteria* phage GEC-3S (1e-58)	97.8	0	N
215	145517-145726	-	69	Hypothetical protein	*Escherichia* phage RB49 (5e-42)	98.6	0	N
216	145746-146084	-	112	Hypothetical protein	*Escherichia* virus KFS-EC (2e-72)	96.4	0	N
217	146084-146344	-	86	Hypothetical protein	*Escherichia* phage ECD7 (1e-53)	97.7	0	N
218	146334-146588	-	84	Hypothetical protein	*Enterobacteria* phage GEC-3S (1e-53)	96.4	0	N
219	146585-147019	-	144	Hypothetical protein	*Shigella* phage JSE (4e-104)	98.6	0	N
220	147030-147227	-	65	Hypothetical protein	*Escherichia* phage JSE (3e-40)	98.5	0	N
221	147238-147528	-	96	Hypothetical protein	*Escherichia* phage RB49 (2e-63)	100	0	N
222	147630-148406	-	258	Receptor-recognizing protein<br>[PF05268; IPR007932; Receptor-recog_Gp38; Receptor-recognising protein Gp38]	*Escherichia* phage JSE (2e-173)	98.5	0	Y
223	148461-	-	73	Hypothetical protein	*Escherichia* phage RB49 (2e-44)	98.6	0	N

	148682							
224	148772-149056	-	94	Hypothetical protein	*Escherichia* phage Phi1 (3e-60)	94.7	0	N
225	149053-149403	-	116	Hypothetical protein	*Escherichia* phage JSE (3e-77)	100	0	N
226	149400-149738	-	112	Hypothetical protein	*Escherichia* phage ECD7 (2e-77)	98.2	0	N
227	149728-149937	-	69	Hypothetical protein	*Escherichia* phage ECD7 (1e-40)	100	0	N
228	150265-150663	-	132	Hypothetical protein [PF19174; IPR043876; DUF5856; Protein of unknown function DUF5856]	*Escherichia* phage ECD7 (5e-91)	97.7	0	N
229	150650-150952	-	100	Lysis inhibition	*Escherichia* phage ECD7 (3e-69)	100	0	Y
230	151021-151617	-	198	Thymidine kinase [PIRSF035805; IPR001267; Thymidine_kinase; Thymidine kinase]	*Escherichia* phage JSE (2e-144)	99.5	0	N
231	151617-151910	-	97	Hypothetical protein	*Escherichia* phage RB49 (5e-63)	99	2	N
232	151903-152121	-	72	Hypothetical protein	*Escherichia* phage RB49 (1e-46)	100	0	N
233	152118-152312	-	64	Hypothetical protein	*Escherichia* phage RB49 (3e-39)	98.4	0	N
234	152302-152757	-	151	Phosphatase [SM00506; IPR002589; Macro_dom; Macro domain]	*Enterobacteria* phage GEC-3S (4e-104)	98	0	N
235	152773-153132	-	119	Valyl-tRNA synthetase modifier	*Escherichia* phage RB49 (8e-81)	98.3	0	Y
236	153140-153730	-	196	Hypothetical protein [PF01464; IPR008258; Transglycosylase_SLT_dom_1; Transglycosylase SLT domain 1]	*Escherichia* phage JSE (3e-139)	99.5	0	Y
237	153816-154238	-	140	Endonuclease [PF10715; IPR019653; T4_endoribonuclease_RegB; T4 endoribonuclease RegB]	*Escherichia* phage JSE (4e-99)	100	0	N
238	154243-154578	-	111	Hypothetical protein	*Escherichia* virus KFS-EC (3e-77)	100	0	N
239	154636-154881	-	81	Hypothetical protein	*Enterobacteria* phage GEC-3S (2e-53)	100	0	N
240	154878-155072	-	64	Hypothetical protein	*Escherichia* phage JSE (7e-38)	95.3	0	N
241	155094-155792	-	232	Nucleotidyltransferase [PF10127; IPR018775; RlaP; RNA repair pathway DNA polymerase beta]	*Escherichia* phage RB49 (2e-172)	99.6	0	N
242	155811-156404	-	197	Putative HNH endonuclease [PF07463; IPR010902; NUMOD4; NUMOD4]	*Escherichia* phage vB_EcoM_G37-3 (6e-138)	96.5	0	N

243	156453-156866	-	137	Vs.6 conserved hypothetical protein [PS51149; IPR001150; Gly_radical; Glycine radical domain]	*Escherichia* phage vB_EcoM_G37-3 (1e-79)	88.4	0	N
244	156877-157002	-	41	Hypothetical protein	*Escherichia* phage RB49 (4e-20)	100	0	N
245	157002-157493	-	163	Hypothetical protein	*Escherichia* virus KFS-EC (3e-114)	100	0	N
246	157556-157684	-	42	Hypothetical protein [PS51032; IPR001471; AP2/ERF_dom; AP2/ERF domain]	*Escherichia* phage JSE (4e-21)	100	0	N
247	157684-158130	-	148	Hypothetical protein [PF13392; IPR003615; HNH_nuc; HNH nuclease]	*Escherichia* phage JSE (1e-86)	98.4	0	N
248	158176-158565	-	129	Endolysin [PF13539; IPR039561; Peptidase_M15C]	*Escherichia* phage JSE (1e-89)	100	0	N
249	158594-158785	-	63	Hypothetical protein	*Escherichia* phage RB49 (1e-33)	93.7	2	N
250	158787-159002	-	71	Hypothetical protein	*Escherichia* phage RB49 (4e-43)	98.6	2	N
251	159005-159184	-	59	Hypothetical protein	*Escherichia* phage ECD7 (2e-31)	100	2	N
252	159431-159547	-	38	Hypothetical protein	*Enterobacteria* phage GEC-3S (4e-15)	94.7	1	Y
253	159865-160185	-	106	Hypothetical protein	*Escherichia* virus KFS-EC (2e-69)	99.1	0	N
254	160207-160404	-	65	Hypothetical protein	*Escherichia* phage RB49 (4e-37)	100	2	N
255	160404-160601	-	65	Hypothetical protein	*Escherichia* phage Phi1 (3e-35)	100	2	N
256	160603-160785	-	60	Hypothetical protein	*Escherichia* phage Phi1 (1e-34)	100	2	N
257	160794-161120	-	108	Hypothetical protein [PF18840; IPR041045; LPD25; Large polyvalent protein associated domain 25]	*Escherichia* phage Phi1 (8e-71)	98.2	0	N
258	161200-161388	-	62	Conserved hypothetical protein	*Escherichia* phage JSE (9e-37)	100	0	N
259	161398-161586	-	62	Hypothetical protein	*Enterobacteria* phage GEC-3S (6e-38)	100	0	N
260	161599-161826	-	75	Hypothetical protein	*Escherichia* phage ECD7 (3e-46)	98.7	0	N
261	161893-162078	-	61	Hypothetical protein	*Escherichia* phage RB49 (2e-34)	98.4	0	N
262	162355-162573	-	72	Hypothetical protein	*Escherichia* phage JSE (6e-45)	100	2	N

263	162570-162752	-	60	Hypothetical protein	*Escherichia* phage ECD7 (6e-34)	95	2	N
264	162749-162973	-	74	Hypothetical protein	*Escherichia* phage JSE (1e-46)	100	2	N
265	162970-163080	-	36	Hypothetical protein	*Escherichia* phage RB49 (7e-16)	100	1	N
266	163077-163274	-	65	Hypothetical protein	*Escherichia* phage RB49 (1e-34)	93.9	2	N
267	163264-163467	-	67	Discriminator of mRNA degradation [PF17587; IPR035137; Dmd; Discriminator of mRNA degradation]	*Escherichia* phage Phi1 (2e-39)	98.5	0	N
268	163477-163635	-	52	Hypothetical protein	*Escherichia* phage ECD7 (1e-23)	98.1	2	N
269	163702-164076	-	124	Hypothetical protein	*Escherichia* phage Phi1 (2e-86)	99.2	0	N
270	164187-164651	-	154	Hypothetical protein [SSF55144; IPR009097; Cyclic_Pdiesterase; Cyclic phosphodiesterase]	*Escherichia* phage vB_EcoM_G2494 (1e-108)	98.7	0	N
271	164654-164974	-	106	Tail fiber chaperone	*Escherichia* phage Phi1 (3e-46)	97.5	0	N
272	165007-165663	-	218	Putative dNMP kinase [SSF52540; IPR027417; P-loop_NTPase; P-loop containing nucleoside triphosphate hydrolase]	*Enterobacteria* phage GEC-3S (8e-160)	100	0	N
273	165679-166269	-	196	Tail completion and sheath stabilizer protein [PF06841; IPR010667; Phage_T4_Gp19; Bacteriophage T4, Gp19, tail tube]	*Escherichia* phage RB49 (2e-146)	99.	0	N
