# Supplementary material for: Characterization of Broad Spectrum Bacteriophage vB ESM-pEJ01 and Its Antimicrobial Efficacy Against Shiga Toxin-Producing Escherichia coli in Green Juice
Source: Microorganisms. 2025 Jan 7;13(1):103. doi: 10.3390/microorganisms13010103 (PMC11767321; doi:10.3390/microorganisms13010103)
Supplement: Supplementary file 1 [file microorganisms-13-00103-s001.zip › Supplementary Table S2.pdf]

Supplementary Table S2. Comparison of amino acid identities between STEC phage vB_ESM-pEJ01 and representative *Krischvirus* phages based on three functional ORF groups in the genome.

Group/function	ORF*	Amino acid identity (%)					
		JSE (NC_012740)	KFS-EC (NC_055757)	RB49 (NC_005066)	Phi1 (NC_009821)	GEC-3S (NC_025425)	ECD7 (NC_041936)
Lysis-related protein							
	6	98.5	98.6	98.8	99	98.8	99
	89	97.3	98	97.3	97.3	95.3	98
	90	100	96.2	97.7	97.2	98.1	89.8
	120	98.6	97.7	99.5	99.5	99.5	99
	248	100	91.4	91.4	91.3	91.4	97.1
Tail fiber protein							
	115	98.3	95.7	97.9	96.6	97.6	99.2
	116	97.8	96.5	98.4	99.2	98.6	99.2
	117	94.3	93.7	99.4	99.6	99.6	99.6
	118	78.1	43.8	76.9	70.3	60.4	71.3
Host specificity-related protein							
	222	96.9	96.9	97.2	96.5	84	96.9

*ORFs annotated in STEC phage vB_ESM-pEJ01 genome.
